# Flimma: a federated and privacy-aware tool for differential gene expression analysis

**DOI:** 10.1186/s13059-021-02553-2

**Published:** 2021-12-14

**Authors:** Olga Zolotareva, Reza Nasirigerdeh, Julian Matschinske, Reihaneh Torkzadehmahani, Mohammad Bakhtiari, Tobias Frisch, Julian Späth, David B. Blumenthal, Amir Abbasinejad, Paolo Tieri, Georgios Kaissis, Daniel Rückert, Nina K. Wenke, Markus List, Jan Baumbach

**Affiliations:** 1 Chair of Experimental Bioinformatics, TUM School of Life Sciences, Technical University of Munich, Freising, Germany; 2 Institute for Computational Systems Biology, University of Hamburg, Hamburg, Germany; 3 AI in Medicine and Healthcare, Technical University of Munich, Munich, Germany; 4 Department of Mathematics and Computer Science, University of Southern Denmark, Odense, Denmark; 5 Department Artificial Intelligence in Biomedical Engineering, Friedrich-Alexander University Erlangen-Nürnberg, Erlangen, Germany; 6 CNR National Research Council, IAC Institute for Applied Computing, Rome, Italy; 7 Sapienza University of Rome, Rome, Italy; 8 Klinikum rechts der Isar, Technical University of Munich, Munich, Germany; 9 Biomedical Image Analysis Group, Imperial College London, London, UK; 10 OpenMined, Oxford, UK

**Keywords:** Differential expression analysis, Federated learning, Privacy of biomedical data, Meta-analysis

## Abstract

Aggregating transcriptomics data across hospitals can increase sensitivity and robustness of differential expression analyses, yielding deeper clinical insights. As data exchange is often restricted by privacy legislation, meta-analyses are frequently employed to pool local results. However, the accuracy might drop if class labels are inhomogeneously distributed among cohorts. *Flimma* (https://exbio.wzw.tum.de/flimma/) addresses this issue by implementing the state-of-the-art workflow *limma voom* in a federated manner, i.e., patient data never leaves its source site. *Flimma* results are identical to those generated by *limma voom* on aggregated datasets even in imbalanced scenarios where meta-analysis approaches fail.

**Supplementary Information:**

The online version contains supplementary material available at (10.1186/s13059-021-02553-2).

## Background

The identification of differentially expressed genes or transcripts, e.g., in diseases or in response to treatment, is a standard but important task in molecular systems medicine.

Differential gene expression analysis compares the expression profiles of two or more groups of samples to reveal genes with significant differences between the groups. Technologies for high-throughput gene expression profiling include microarrays and RNA sequencing, the latter being more widely used in clinical research today. Both are intrinsically different, e.g., signal- vs. count-based measurement, and their results subject to platform-specific biases [1, 2]. Many bioinformatics tools for identifying differentially expressed genes from such data have been developed [3–9]. These methods differ with respect to the assumptions about data distribution (e.g., normal vs. Poisson or negative binomial distribution), the data normalization strategies, and in the test statistic used to detect differentially expressed genes [10–12].

One major challenge of differential expression studies is the lack of robustness due to the high technical and biological variability of the data [13–15], which can be addressed using various strategies [16–18]. The simplest and most effective way would be to increase the sample size [16], which is non-trivial, as data collection is expensive and time-consuming, sample availability may be limited (e.g. metastatic cancer or healthy tissue samples are difficult to obtain), or because existing data can not be shared and pooled as they are subject to personal data protection laws. The latter is of particular concern for next-generation sequencing data, from which the sample donor can be identified under certain conditions [19–21]. Although several human-derived expression profiles are nowadays publicly available, their utility (in particular in clinical settings) is often still limited for inherent privacy issues. The statistical analysis of expression data may require relevant clinical metadata, e.g., patient sex, age, weight, ethnicity, and disease status, which may be identifying when combined. In addition, recent works suggest that patient genotypes can be predicted from RNA-seq data, making patients identifiable through expression profiles or eQTL data obtained from open-access sources [22–24]. Schadt et al. have shown that genotypes can be inferred from expression levels of eQTL-controlled genes and sensitive information — for instance, medical history, phenotypic traits, and family relationships can be revealed [22]. Matching predicted genotypes to known ones allowed for identifying individuals with an accuracy of up to 99% in optimal settings, i.e., when the microarray platform, tissue type, and ancestry were the same for expression and eQTL datasets. Harmanci and Gerstein proposed a measure of individual-characterizing information leakage and investigated its dependence on genotype predictability given the expression dataset [23]. They developed a framework to assess privacy risks before publishing the data. They have also presented a simplified but effective attack scheme where homozygous genotypes were predicted from extreme gene expression values.

To control the exchange of sensitive molecular profiling data from, e.g., next-generation sequencing experiments, databases, such as dbGaP [25] or EGA [26], restrict access to authorized users affiliated with organizations willing to guarantee the legal and secure use of personal data. Nevertheless, the application procedure needs to be repeated per study and per database, making this a difficult and time-consuming process, which is also error-prone if a priori unknown confounder variables are not requested and can thus not be corrected for in the downstream analysis. Alternatively, when direct access to raw data is not possible, researchers can combine the results of several studies using meta-analysis techniques such as Fisher’s method [27], Stouffer’s method [28], RankProd [29], or the random effects model [30] (REM). Meta-analysis is widely adopted for aggregation of genome-wide association studies (GWAS) [31] and differential gene expression analysis results [32, 33] (cf. the “Meta-analysis approaches” section in the “Methods” section for details). The main disadvantage of meta-analysis tools is that their underlying assumptions about the distribution of *p*-values or effect sizes may not be realistic. Furthermore, meta-analysis largely ignores possible differences between cohorts (e.g., class imbalance or heterogeneity of covariate distributions) [34] or data processing steps (e.g., normalization) [35], which may have a significant impact on the results [34].

Privacy-aware techniques, such as federated learning (FL) [36], differential privacy (DP) [37], homomorphic encryption (HE) [38], and secure multi-party computation (SMPC) [39], have recently moved into the focus of research for tasks involving privacy-sensitive patient data [40]. Note that in this paper, the term *privacy-aware* [41] designates the techniques that avoid sharing raw personal data between collaborating parties. Such approaches are usually not in conflict with privacy legislation and may thus legally and practically be applied to real-world medical data. We call such a privacy-aware approach *privacy-preserving* (e.g., DP [37]), if it provides a formally proven privacy guarantee that captures the risks associated with each sample of the dataset.

FL has become increasingly popular in bioinformatics for GWAS [42, 43], survival analysis [44], and additional challenges in patient data processing [40, 45]. FL implies collaborative model training by multiple participants without disclosing private data to any other party [36]. Instead, each participant only shares intermediate model parameters while keeping the private data in the local environment (e.g. the legally safe harbors of the hospitals’ IT system). The local parameters from the clients are aggregated at the server iteratively to compute a globally optimal model.

DP perturbs the data or results by adding noise to them. Although DP is privacy-preserving and complementary to FL, it might dramatically impact the accuracy of the results. HE performs computation on the encrypted data from the participants. It suffers from two practical disadvantages [46]: it supports a limited number of operations such as addition and multiplication, and consequently, it requires approximations to compute non-linear operations (e.g., computing the inverse of covariance matrix in gene expression analysis), leading to accuracy loss in the final results. More importantly, it is computationally expensive because a single machine performs operations on a large amount of encrypted data and might require a sizable amount of memory to process large datasets [47]. In SMPC, each participant computes secret shares from the data and shares them with the computing parties. The computing parties calculate the intermediate results and exchange them among each other to compute the final results. Because SMPC-based methods send secret shares of the data from the participants to computing parties, they consume a huge amount of network traffic [48].

FL is a promising alternative to SMPC and HE in terms of performance and scalability. Unlike HE, it does not increase computational cost much compared to the centralized method, and unlike SMPC, it transfers only a small number of model parameters through the network. Similar to HE, SMPC, and meta-analyses, FL is not privacy-preserving like DP, i.e., the server might reconstruct the raw data using the model parameters obtained from the clients [49–51]. However, the approaches based on pure HE and SMPC provide stronger privacy compared to FL-based approaches because they reveal less information to the third parties.

The privacy of federated methods can be enhanced by applying HE or SMPC on the shared model parameters. In comparison to purely SMPC- or HE-based methods, hybrid approaches are computationally efficient because heavy computations are distributed across the clients. Additionally, they offer enhanced privacy compared to pure FL, because the original values of the local parameters remain hidden from the server, and only global parameters are revealed to the server and the clients.

In this paper, we introduce *Flimma* (federated *limma*), a novel federated privacy-aware tool for the identification of differentially expressed genes called *Flimma*. Our new tool represents a federated implementation of the popular differential expression analysis workflow *limma voom* [52], one of the standard pipelines widely applied in the field for expression analyses. We have chosen *limma voom* among other popular count-based methods, because it is comparably fast without sacrificing accuracy [5]. Besides that, *Flimma* could be easily modified for handling microarray data, since the *limma* method was originally designed for such data [53] and only later extended to RNA-seq data via *voom* [5].

**Fig. 1:**
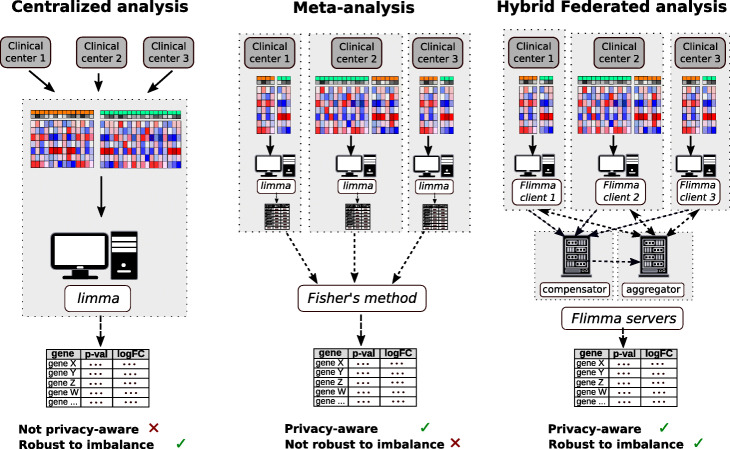
Gene expression analysis in case of multi-center studies. Bold arrows show the exchange of raw data, dashed arrows – the exchange of model parameters or summary statistics. Grey areas highlight different physical locations

*Flimma* is based on *HyFed* [54], a hybrid FL framework, which applies additive secret sharing-based SMPC method to avoid disclosing the local model parameters to the server (see the “Methods” for details). It provides several advantages over the existing approaches for gene expression analysis (Fig. 1). Unlike *limma voom*, *Flimma* enhances the privacy of the data in the cohorts since the expression profiles never leave the local execution sites and only aggregated parameters are revealed to the server and the other local sites. In contrast to meta-analysis approaches, *Flimma* is particularly robust against heterogeneous distributions of data (in particular of confounders and class labels) across the different cohorts, which makes it a powerful alternative for multi-center studies where patient privacy is a key concern.

## Results

We applied *Flimma* and four meta-analyses approaches on two real-world datasets: a breast cancer expression dataset from TCGA [55] and a skin dataset from GTEx [56]. To assess *Flimma*’s power, we model the multi-party setting by randomly partitioning both datasets into virtual cohorts, while introducing different levels of imbalance w.r.t. target class labels and covariate distributions. For both datasets, we simulated three realistic scenarios leading to different levels of sample distribution heterogeneity between local cohorts. We split the breast cancer dataset such that three virtual cohorts yield different frequencies of the LumA subtype to simulate an imbalanced distribution of disease subtypes collected at different clinical centers (Table 1). Inaddition, we partitioned the TCGA breast cancer dataset according to tissue source sites. Similarly, GTEx skin dataset was split by the mean ischemic time to illustrate the effect of potential confounders such as differences in sample collection and/or processing between the participating laboratories (Table 2).

**Table 1:** Characteristics of three scenarios for the TCGA-BRCA dataset. The distributions of ages and tumor stages were balanced

	Cohort sizes			Frequency of basal subtype			Frequency of LumA subtype		
	Cohort 1	Cohort 2	Cohort 3	Cohort 1	Cohort 2	Cohort 3	Cohort 1	Cohort 2	Cohort 3
No imbalance	283	283	284	0.20	0.20	0.20	0.57	0.57	0.58
Mild imbalance	121	242	487	0.10	0.30	0.17	0.40	0.50	0.66
Strong imbalance	65	196	589	0.25	0.50	0.09	0.14	0.50	0.65

**Table 2:** Characteristics of the scenarios for the GTEx skin dataset. The frequencies of samples obtained from male and female individuals were similar in all cohorts (between 30 and 34% samples from females in all scenarios)

	Cohort sizes			Fraction of sun-exposed skin samples			Mean ischemic time, min		
	Cohort 1	Cohort 2	Cohort 3	Cohort 1	Cohort 2	Cohort 3	Cohort 1	Cohort 2	Cohort 3
No imbalance	425	425	427	0.53	0.53	0.53	629	636	636
Mild imbalance	181	363	733	0.4	0.65	0.51	490	620	676
Strong imbalance	97	293	887	0.8	0.4	0.54	347	646	661

We then compared *Flimma* with popular meta-analysis tools using the *limma voom* results on the pooled datasets as gold standard. In summary, *Flimma* obtained the same results as *limma voom* in all tests. Across all experiments, the maximal absolute difference for log-transformed *p*-values and log-fold-change values computed by *Flimma* and *limma voom* did not exceed 0.1 (Additional file 1: Table S1). In contrast, the results of the meta-analysis methods diverged from the results of *limma voom*, and this effect was especially pronounced in imbalanced scenarios.

One of the main pitfalls of gene expression analysis is the presence of strong batch effects in the data. Even for technical replicates, gene expression levels measured in two laboratories may drastically differ due to the difference in sample preparation and library construction protocols, sequencing platforms, chemical reagents, and many other known and unknown experimental factors. To demonstrate that *Flimma* is robust to experimental batch effects, we applied it to three independent breast cancer datasets generated at different laboratories.

### Evaluation on artificial dataset splits

We compared negative log-transformed *p*-values computed by all privacy-aware approaches (i.e., *Flimma* and meta-analysis methods) with the results obtained by running *limma voom* on the combined dataset. For the privacy-aware approaches, we computed the root mean square error (RMSE), the precision, the recall, the F1 score, the Pearson and the Spearman correlation w.r.t. the results of the aggregated analysis with *limma voom*, which we treated as ground truth.

**Fig. 2:**
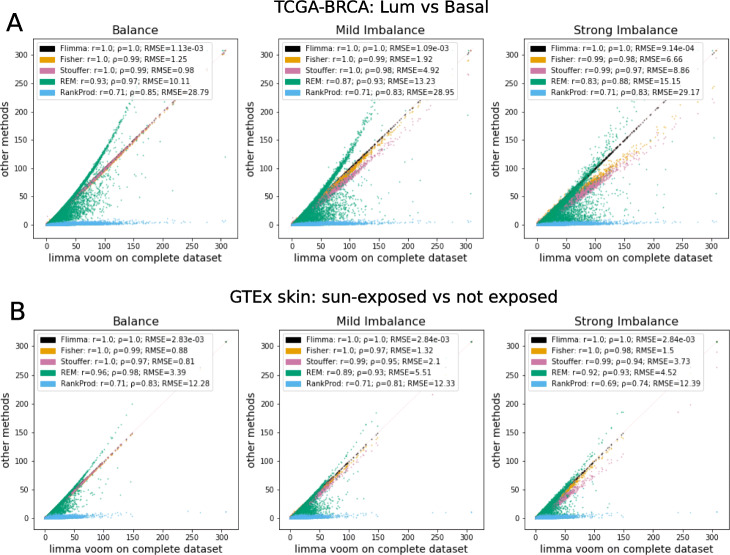
The comparison of negative log-transformed *p*-values computed by *Flimma* and meta-analysis methods (*y*-axis) with *p*-values obtained by *limma* on the aggregated dataset (*x*-axis) in three scenarios on **A** TCGA-BRCA and **B** GTEx skin datasets. Pearson correlation coefficient (r), Spearman correlation coefficient (*ρ*), and root-mean squared error (RMSE) calculated for each method are reported in the legend

As shown in Fig. 2, Tables 3-4, Additional file 2: Table S2, and Additional file 3: Table S3, *Flimma* produces the same p-values as the aggregated analysis with *limma voom* in all scenarios, including the imbalanced ones. This implies that *Flimma* is robust against heterogeneous data distributions across the clients. However, this is not the case for the meta-analysis approaches. In general, their RMSEs increase (and Pearson correlations decrease) as the scenarios become more imbalanced, and they introduce false positives and false negatives even in the balanced scenario. In spite of the difference in *p*-values calculated by all meta-analysis methods, their gene rankings were quite similar to the ranking produced by the aggregated *limma voom* (the Spearman correlation varied between 0.74 to 0.99 in all experiments).

**Table 3:** F1 score, the number of false positives (FP) and the number of false negatives (FN) obtained on TCGA-BRCA dataset in three scenarios. Values corresponding to the best performance over all methods are italicized. All calculated performance measures are reported in Additional file 2: Table S2

	F1			FP			FN		
Scenario	Balanced	Mildly imbalanced	Strongly imbalanced	Balanced	Mildly imbalanced	Strongly imbalanced	Balanced	Mildly imbalanced	Strongly imbalanced
Flimma	*1.00*	*1.00*	*1.00*	*0*	*0*	*0*	*0*	*0*	*0*
Fisher	*1.00*	0.92	0.93	14	248	192	8	290	265
Stouffer	*1.00*	0.92	0.93	14	245	189	9	290	265
REM	*1.00*	0.97	0.95	12	80	121	17	119	215
RankProd	*1.00*	0.92	0.93	14	243	193	12	295	274

**Table 4:** F1 score, the number of false positives (FP), and the number of false negatives (FN) obtained on GTEx skin dataset in three scenarios. Values corresponding to the best performance over all methods are italicized. All calculated performance measures are reported in Additional file 3: Table S3

	F1			FP			FN		
Scenario	Balanced	Mildly imbalanced	Strongly imbalanced	Balanced	Mildly imbalanced	Strongly imbalanced	Balanced	Mildly imbalanced	Strongly imbalanced
Flimma	*1.00*	*1.00*	*1.00*	*0*	*0*	*0*	*0*	*0*	*0*
Fisher	0.99	0.91	0.83	4	32	67	*0*	18	33
Stouffer	0.99	0.91	0.83	4	32	67	*0*	18	33
REM	0.99	0.95	0.94	4	15	21	2	14	12
RankProd	0.99	0.91	0.83	4	32	67	*0*	18	33

### Performance for top-ranked genes

**Fig. 3:**
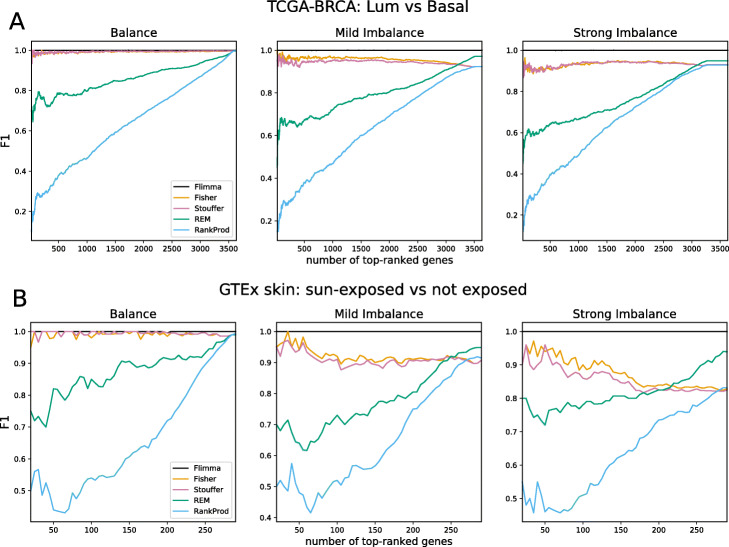
The dependency of the F1 score on the number of top-ranked genes considered to be differentially expressed. Genes were ranked in order of their negative log-transformed *p*-values decreasing and the number of top-ranked genes varied between 20 and 3500 (for TCGA-BRCA dataset, **A**) and 300 (for GTEx Skin dataset, **B**) with step 5

Since some research tasks such as biomarker discovery require the identification of a small number of significantly differentially expressed genes, we investigated how the performance of the methods varies with altered numbers of selected top differentially expressed genes after sorting by *p*-value (Fig. 3 and Additional file 4: Figures S1-2). Again, *Flimma* perfectly reproduced the results of aggregated *limma voom* in all scenarios and outperformed all meta-analysis approaches. Fisher’s and Stouffer’s methods demonstrated almost perfect performance in the balanced scenario, but their performance decreased in the imbalanced ones.

### Splitting TCGA-BRCA by sample source site

TCGA is a multi-center project and tumor samples of TCGA-BRCA datasets were collected at 37 different clinical centers, which can result in some between-center variability. Therefore, we also evaluated *Flimma* and its baselines on a more realistic scenario, where TCGA-BRCA dataset was split according to the sample source sites, but we kept only 14 of the 37 cohorts, such that each cohort contained at least 3 samples of LumA and basal subtype.

We selected 3, 5, 7, 10, and 14 cohorts such that subtype frequencies, mean stage, and age are dissimilar across the selected cohorts (cf. Additional file 5: Table S4 for details). We also added additional terms in linear models to account for possible cohort effects. Similar to the previous experiments, *Flimma* clearly outperforms all meta-analysis approaches in terms of RMSE, precision, and recall (Table 5 and Additional file 6: Table S5).

**Table 5:** RMSE, precision, and recall obtained by *Flimma* and the meta-analysis tools on TCGA-BRCA datasets split by tissue source sites

The number of cohorts	3	5	7	10	14
RMSE					
Flimma	*0.0008*	*0.0007*	*0.0008*	*0.0017*	*0.0012*
Fisher	0.94	1.82	2.53	3.86	5.37
Stouffer	1.47	2.21	2.87	4.26	5.68
REM	2.73	3.68	4.75	7.21	8.50
RankProd	5.16	8.19	11.32	18.92	23.50
Precision					
Flimma	*1.00*	*1.00*	*1.00*	*1.00*	*1.00*
Fisher	0.85	0.88	0.90	0.93	0.95
Stouffer	0.85	0.88	0.91	0.93	0.95
REM	0.93	0.94	0.95	0.97	0.97
RankProd	0.92	0.87	0.90	0.93	0.95
Recall					
Flimma	*1.00*	*1.00*	*1.00*	*1.00*	*1.00*
Fisher	0.92	0.95	0.95	0.96	0.97
Stouffer	0.89	0.93	0.94	0.96	0.97
REM	0.93	0.96	0.97	0.98	0.98
RankProd	0.87	0.96	0.96	0.96	0.97

Values corresponding to the best performance over all methods are italicized

### Robustness to batch effects

To demonstrate the robustness of *Flimma* towards experiential batch effects, we applied it on three additional publicly available breast cancer cohorts from GEO: GSE129508 [57], GSE149276 [58], and GSE58135 [59]. These datasets were independently collected and sequenced at three different laboratories and subjected to various experimental biases related to sample preparation, library construction, and sequencing platform (Additional file 7: Table S6). However, we assumed that collaborating partners can agree to use the same quantification pipeline and therefore obtained uniformly (in silico) preprocessed raw read counts from ARCHS^4^ [60].

**Fig. 4:**
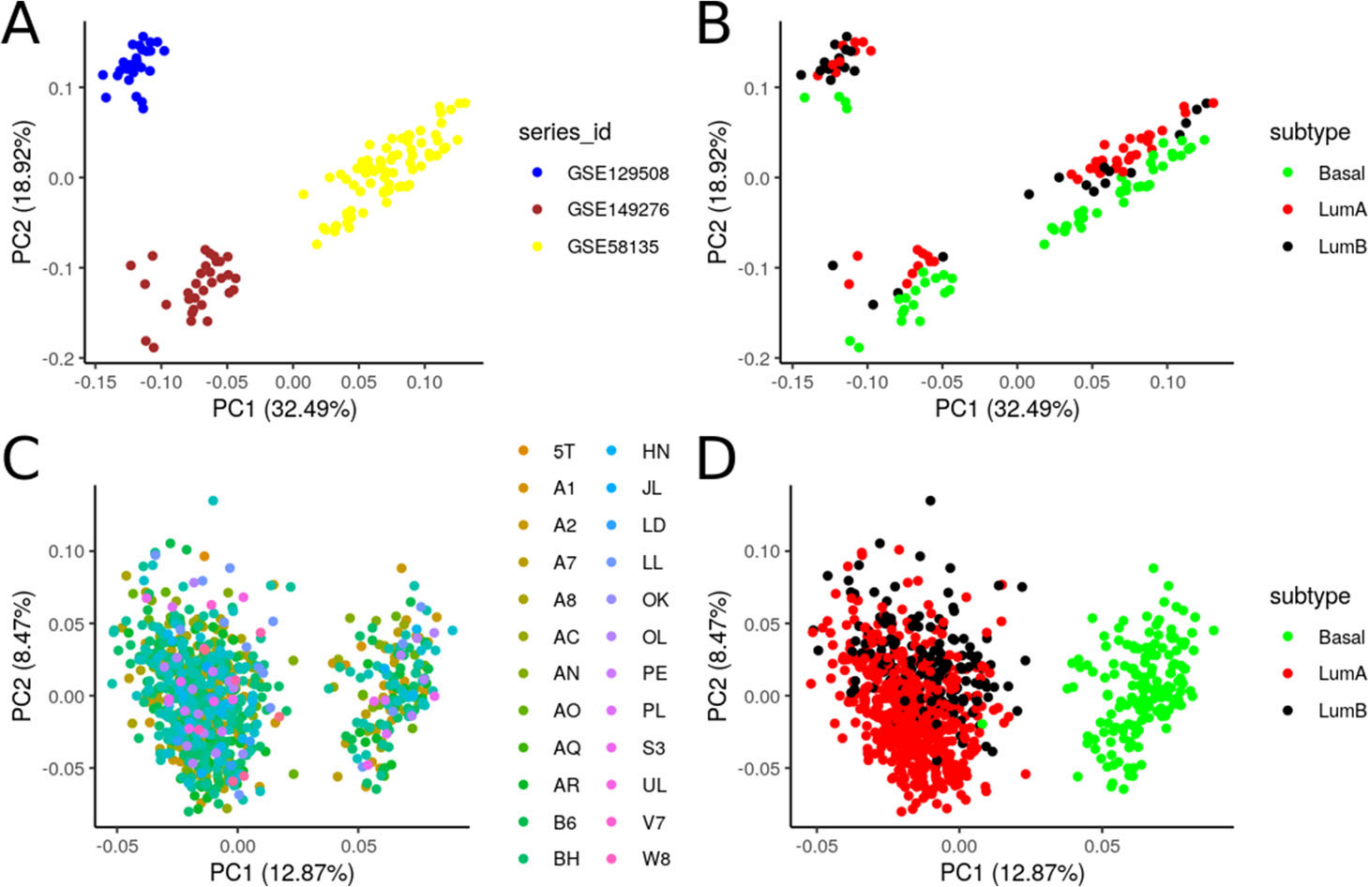
PCA projections computed and plotted by *proBatch* R package [99] of samples from three GEO cohorts (**A**, **B**) and TCGA-BRCA cohorts (**C**, **D**) colored according to cohort (**A**, **C**) and cancer subtype (**B**, **D**)

In contrast to TCGA-BRCA, cohort-specific batch effects in the GEO datasets were much more pronounced. Principal component analysis revealed that the differences between samples from different cohorts were much larger than the differences between subtypes within the same cohort (Fig. 4). In this case, effective adjustment for batch effect before testing for differential expression is crucial [61]. This can be done in two ways, either via subtracting the variation explained by batch from the data or via the inclusion of additional variables accounting for batch effects to the model. With *Flimma*, we implemented the second approach, as it is preferable for downstream statistical analysis [62]. Below, we will demonstrate that this approach effectively handles the batch effects in our breast cancer data sets and gives almost identical results. Several methods for batch effect correction exist, but not all of them are compatible with *limma voom* because the latter is computing count-based statistics. A recently published modification of the state-of-the-art batch-effect correction method *ComBat* [63], namely *ComBat-Seq* [64], is developed specifically to handle read count data. Hence, we utilized the results of *limma voom* obtained on the centralized GEO cohort after the removal of laboratory-specific effects by *ComBat-Seq* as a gold standard in the following experiments.

**Fig. 5:**
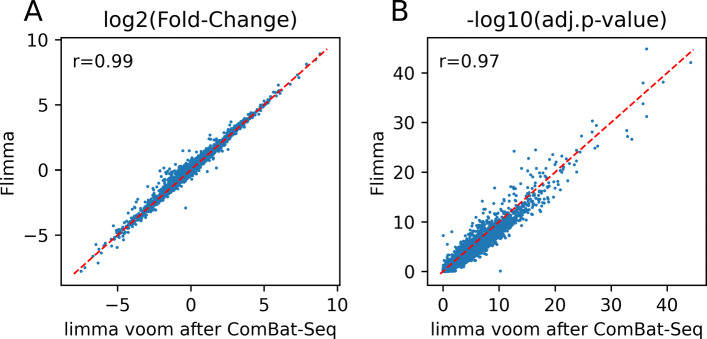
Comparison of the results obtained by *Flimma* on uncorrected GEO data with the results of *limma voom* after batch effect removal by *ComBat-Seq*

In *Flimma*, we model the batch effects of datasets by adding *m*−1 binary covariates to the linear model, where *m* is the number of datasets. Despite the strong batch effects in the GEO data, *Flimma* returned nearly the same fold-changes and BH-adjusted *p*-values as *limma voom* run on the same data after batch effect removal by *ComBat-Seq* (Fig. 5). Moreover, our results suggest that the approach used by *Flimma* gives better results than batch effect correction based on one or several first principal components (Additional file 4: Supplementary Text and Additional file 8: Table S7).

## Discussion

In this work, we presented *Flimma*, a privacy-aware tool for differential expression analysis. While *Flimma* results are mathematically equivalent to *limma voom*, *Flimma* can operate on distributed cohorts without the disclosure of sensitive data. To enhance data privacy, *Flimma* uses a hybrid federated approach, where the local parameters of the clients are hidden from the server and only global parameters resulting from the aggregation are disclosed. We employed *HyFed* to implement *Flimma* because unlike similar methods such as [65], it is an open source framework with a Python API (application programming interface) to develop hybrid federated tools. Moreover, it supports federated mode, in which different components can securely communicate over the Internet using the HTTPS protocol.

In this work, we have demonstrated that *Flimma* is superior to meta-analyses in imbalanced scenarios when the distributions of class labels or covariates are not identical between cohorts. We have also shown that *Flimma* is robust to technical batch effects.

One limitation of this work is the absence of a gold standard for the evaluation of differential expression analysis results. ABCD mixtures used in RNA-seq benchmark projects [1, 66] are not suitable for this study, since only five or less replicates of each mixture are sequenced by each participant. Although these projects are multi-center studies, such a small number of samples per participating center would not be realistic for mimicking modern biomedical studies involving human patients. Moreover, with these artificial mixtures, we could not model biological variability which is intrinsic of real-world patient-derived data. Therefore, we have only tested *Flimma* on patient-derived expression datasets, split them into parts modeling independent cohorts if necessary and considered the results of *limma voom* obtained on the combined datasets as ground truth.

### Remaining privacy risks

Although *Flimma* greatly enhances data privacy compared to centralized analysis, it does not provide a perfect privacy guarantee which quantifies the risk associated with the individual samples in the dataset. *Flimma* assumes non-colluding parties, e.g., the aggregator or compensator never exchanges the individual noisy parameters or noise values from the clients with each other, and there are more than two clients participating in the study. Another assumption is honest-but-curious parties, which stick with the protocol and follow it but try to reconstruct the data from the model parameters.

One possible scenario of such a reconstruction attack is the recovery of the global *X* from *X*^*T*^*X* by the aggregator, if the number of samples is close to the number of covariates. However, this is not realistic for differential expression analysis because the former should be much larger than the latter for a reliable analysis.

Another potential threat is the presence of a column with all 0 but one 1 in the global design matrix *X*. In this case, *X*^*T*^*Y* reveals the expression profile of a sample with a non-zero value in that column. This is also an unlikely scenario because covariate columns that contain just a single non-zero element are not informative for differential expression analysis and should not be included in the model.

Since it is impossible to oversee all potentially possible scenarios where reconstruction might be feasible, the users should be aware that *Flimma* cannot fully exclude the risk of reconstruction attacks at intermediate results. Providing a privacy guarantee using DP to capture the privacy risks of patients in the dataset while preserving the accuracy of the results in a satisfactory level remains the direction for future research. Note that the risk of reconstruction attack is not excluded for meta-analysis methods. Although local *p*-values and effect sizes appear to be less prone to reconstruction attack than the aforementioned intermediate global parameters computed by *Flimma*, no formal proof of this intuition is provided. Despite that, meta-analyses remain popular approaches that are not in conflict with privacy legislation. In addition to a reasonable protection of the raw data, *Flimma* offers better accuracy than meta-analysis methods.

### Future directions

While *limma voom* is a state-of-the-art method for differential expression analysis that performs favorably in benchmarks [5], other methods for normalization (e.g., quantile normalization [67]) and differential expression analysis (such as *edgeR* [3], *DESeq2* [6], or *sleuth* [9]) exist and may yield different results depending on the dataset used. We thus consider extending *Flimma* with federated implementations of alternative methods in the future.

Another prospective direction for future work is the development of accessory tools for gene expression analysis. This includes for example, federated principle component Analysis (PCA), useful for quality control, or federated batch effect correction methods, such as *ComBat* or *RUVSeq* [68]. Although we have shown that the current version of *Flimma* effectively handles batch effects, other analyses of expression data such as clustering or classification might require transformed data.

Although *limma* has been initially developed for differential gene expression analysis, it is widely used for the analysis of various omics data types, e.g., proteomics [69, 70], metabolomics [71], and microbiomics [72]. Therefore, we plan the development of *Flimma* modifications suitable for the analysis of other omics data types in the future.

## Conclusions

*Flimma* is a privacy-aware tool for the federated identification of differentially expressed genes. It is user-friendly and publicly available at https://exbio.wzw.tum.de/flimma/including tutorials and a video documentation on its principle and application to real data. While *Flimma* results are mathematically equivalent to *limma voom*, *Flimma* operates on distributed cohorts without the disclosure of sensitive data. To enhance data privacy, *Flimma* uses a hybrid federated approach, where the local parameters of the clients are hidden from the server and only global parameters resulting from the aggregation are disclosed. In contrast to meta-analysis approaches, *Flimma* is robust against heterogeneous distribution of data across the different sites and to technical batch effects. In summary, *Flimma* is a promising alternative to meta-analysis methods for multi-center gene expression projects, as it enhances patient privacy while providing the same results as a centralized analysis.

## Methods

### The *limma voom* workflow

*limma voom* is the state-of-the-art method for differential expression analysis. Initially designed for microarrays [53], it was extended by the *voom* function, which removes the mean-variance trend from RNA-seq data and makes it suitable for analysis by *limma* [5]. Recently, the authors of *limma* published an updated guideline on the recommended *limma voom* workflow [52]. Data preprocessing steps of this workflow include removal of weakly expressed genes using the *filterByExpr* function from the *edgeR* package, conversion of raw read counts to log2-transformed counts per million (log-CPM), and normalization of gene expression distributions. We only differ from this workflow by using the upper-quartile (UQ) normalization [35] instead of the trimmed mean of M-values (TMM) normalization [73], since the latter would require disclosing one of the sample profiles to all participants. Although UQ is not the only normalization method that could be implemented in a federated fashion, we have chosen it because it is one of the most widely used in the field [68, 74]. Since no normalization method outperforms others in all cases [75, 76], we are going to implement more federated normalization methods in the future. Furthermore, given the matrix of normalized log-CPM values and the design matrix, *voom* computes precision weights, which compensate for the mean-variance bias that is typical for RNA-seq data and thus makes them suitable for use in *limma*.

### *Flimma*

#### Implementation

**Fig. 6:**
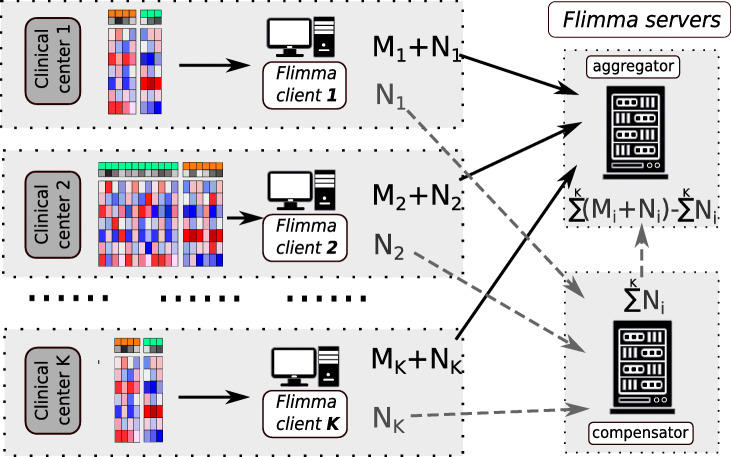
The scheme of *Flimma*. *M* denotes local intermediate parameters, *N* denotes local noise. *K* is the total number of participants. Note that addition and subtraction may be ordinary or modular, see the “Masking scheme” section for details

*Flimma* is based on *HyFed* (https://github.com/tum-aimed/hyfed) [77], a hybrid federated framework implementing an SMPC-like approach to hiding the original values of the local parameters from the server (Fig. 6) [54]. *HyFed* comprises four software components: an aggregator server, a compensator server, a client app, and a web interface.

To start the project, the coordinating user signs into the web interface, creates the project, sets its parameters (e.g., confounding factors, etc.), and invites the participants. Each participant receives a token and a project ID from the coordinator and locally runs the client app to join the study and to select the local dataset. The computations are orchestrated by the aggregator server, which coordinates the clients, aggregates their local model parameters to global parameters, and returns global parameters to clients. Unlike in FL, with *HyFed*, clients mask their local parameters with noise before sending them to the aggregator to enhance the data privacy. The noise matrix has the same shape as the parameter matrix and contains random numbers. The approach to random number generation depends on the data type of the masked matrix and is described in detail in the next section. The noise matrix is sent to the compensator server, which aggregates the noise received from all clients and shares the global noise matrix with the aggregator. The aggregator calculates noisy global parameters and denoises them, by subtracting the global noise matrix provided by the compensator from the noisy global parameters. The proposed hybrid approach provides improved privacy, because a reconstruction attack would require compromising two servers in this case. The aggregator and compensator server components should run in separate machines at distant physical locations. Ideally, to minimize the risk of reconstruction attacks, they should be controlled by third-party organizations not connected to any of the study participants. Currently, the publicly available *Flimma* web tool is using the aggregator running at the Chair of Experimental Bioinformatics, Technical University of Munich (Germany), while the compensator is hosted at the Department of Mathematics and Computer Science at the University of Southern Denmark (Denmark).

As the original *limma voom*, each *Flimma* client accepts a matrix of read counts and a design matrix, specifying class labels and covariates for each sample. *Flimma* outputs a table with *p*-values, fold-changes, and moderated *t* statistics for each gene.

*Flimma* is publicly available at https://exbio.wzw.tum.de/flimma/. The “HowTo” page provides a quick-start guide for *Flimma* along with test data and describes input file formats.

#### Masking scheme

*Flimma* employs the local parameter masking approach of *HyFed*, which treats non-negative integer-valued parameters and real-valued parameters differently. For masking non-negative integers, it applies the standard additive secret sharing scheme based on modular arithmetic over the finite field $\mathbb {Z}_{p}=\{0, 1, p-1\}$, where *p* is a prime number [39]. The elements of noise matrix *N*_*i*_ are drawn from $\mathbb {Z}_{p}$ and added to parameters matrix *M*_*i*_ using modular addition over $\mathbb {Z}_{p}$, i.e., $M^{\prime }_{i}= (M_{i}+ N_{i}) \mod p$. The compensator and aggregator also use modular addition to compute global noise $N = \left (\sum \limits _{i=1}^{K} N_{i} \right) \mod p$ and global noisy parameters $M^{\prime } = \left (\sum \limits _{i=1}^{K} M^{\prime }_{i}\right) \mod p$. Finally, the aggregator removes the global noise from global noisy parameters *M*=(*M*^′^−*N*) mod *p*.

Real-valued parameters are protected by the secret sharing approach based on Gaussian distribution [78, 79]. Noise values are drawn from $\mathcal {N}\left (0,\sigma ^{2}\right)$ added to local parameters: $M^{\prime }_{i} = M_{i} + N_{i}$. Noise aggregation and compensation is performed using ordinary addition and subtraction operations, respectively: $N = \sum \limits _{i=1}^{K} N_{i}$, $M = \sum \limits _{i=1}^{K} M^{\prime }_{i} - N$.

The theoretical analysis of information leakage for additive secret sharing based on modular arithmetics [39] and the real value secret sharing based on Gaussian distribution [79] using the mutual information criterion [80] are provided in the literature [54]. The mutual information measures the reduction in uncertainty about one random variable (e.g., the original values of local parameters *M*_*i*_) given the knowledge of another random variable (e.g., noisy local parameters $M^{\prime }_{i}$). Regarding the original and noisy local parameters with non-negative integer values, it has been shown that the mutual information between them is zero, and thus, the noisy local parameters leak no information about the original local parameters [39]. For real-valued local parameters, however, the upper-bound on mutual information between *M*_*i*_ and $M^{\prime }_{i}$ is: $\frac {1}{2}\log _{2}{\left (1+\frac {\sigma ^{2}_{M_{i}}}{\sigma ^{2}}\right)}$, where $\sigma ^{2}_{M_{i}}$ and *σ*^2^, indicate the variance of the original values of the local parameters and the variance of the Gaussian noise, respectively. That is, the maximum amount of information about *M*_*i*_ disclosed by $M^{\prime }_{i}$ depends on $\frac {\sigma ^{2}_{M_{i}}}{\sigma ^{2}}$.

In practice, *Flimma* sets *p* equal to 2^54^−33, the largest prime number that can fit in a 54-bit integer, and *σ*^2^=10^12^, which is large enough for typical gene expression from the privacy perspective. The mean of the Gaussian noise generator has no significant impact on privacy [79], and therefore, *Flimma* sets it to zero. To ensure the correctness of the results for non-negative local parameters, overflow must not occur during the computation of the aggregated noise, aggregated noisy local parameters, and $ \sum \limits _{i=1}^{K} M_{i} < p$. The value of *p* can be set to larger values to support larger integers but at the cost of supporting a fewer number of clients [54]. Likewise, too large values of *σ*^2^ might impact the precision of the results. However, we confirmed that with default values of *p* and *σ*^2^, the differences between *p*-values and *t* statistics computed by *Flimma* with and without masking the local parameters never exceeded the 10^−8^.

#### Workflow

**Fig. 7:**
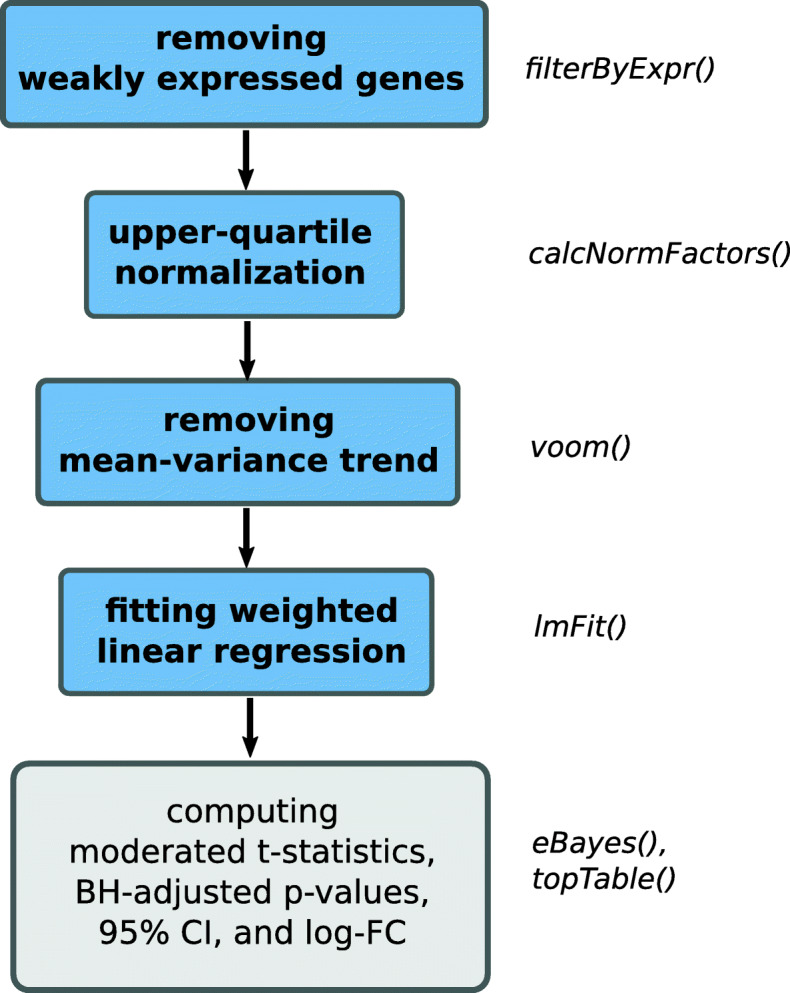
The scheme of *Flimma* workflow. Steps that were reimplemented in a federated fashion are shown in blue. The names of the functions used in the *limma voom* workflow are shown on the right of the flowchart

*Flimma* implements a federated version of the *limma voom* workflow, allowing privacy-aware detection of differentially expressed genes. The scheme of the *Flimma* workflow is presented in Fig. 7.

First, genes that do not have sufficient counts for further statistical analysis are removed. For this, we implemented a federated version of the *filterByExprs* function [81] from the *edgeR* package, which employs two filters: *min*_*total*_*count* filter and CPM cutoff. The first filter removes genes whose sum of counts over all samples does not exceed *min*_*total*_*count* threshold. The second filter excludes genes expressed in insufficient number of samples. It keeps only genes where at least *min*_*n*_*samples* samples pass the CPM cutoff. This cutoff is calculated as a ratio of *min*_*count* over the median library size multiplied by 10^6^, where *min*_*n*_*samples* is defined by the smallest group size in the design matrix. The function parameters *min*_*count* and *min*_*total*_*count* are set to 10 and 15 by default and can be adjusted by the user.

UQ normalization performed in the second step of the pipeline requires the exchange of scaled normalization factors which cannot be used to reveal any private data. The third and the fourth steps of the workflow resemble the *voom* and *lmFit* functions from the *limma* package, which are fitting linear regression models. For training the linear regression model in the federated fashion, *Flimma* utilizes the same approach described by [82]. For each gene, each of *n* clients compute local noisy results $\left (X^{i}\right)^{T}X^{i} + N^{i}_{X^{T}X}$ and $\left (X^{i}\right)^{T}Y^{i} + N^{i}_{X^{T}Y}$, where *X*^*i*^ is a real-valued design matrix, *Y*^*i*^ is the vector of normalized log2-CPM values for the gene, $N^{i}_{X^{T}X}$ and $N^{i}_{X^{T}Y}$ are the noise matrices, and *i* is the index of a client, and sends them to the server. The compensator summarizes noise from clients to global noise

$$N_{X^{T}X} = \sum\limits_{i=1}^{K}{N^{i}_{X^{T}X}}, N_{X^{T}Y} = \sum\limits_{i=1}^{K}{N^{i}_{X^{T}Y}},$$

and shares it with the aggregator. The aggregator computes global noisy results *X*^*T*^*X* and *X*^*T*^*Y* and denoises them:

$$X^{T}X = \sum\limits_{i=1}^{K}{\left(\left(X^{i}\right)^{T}X^{i} + N^{i}_{X^{T}X} \right)} - N_{X^{T}X},$$

$$X^{T}Y = \sum\limits_{i=1}^{K}{\left(\left(X^{i}\right)^{T}Y^{i} +N^{i}_{X^{T}Y} \right)} - N_{X^{T}Y}.$$

The denoised *X*^*T*^*X* and *X*^*T*^*Y* are used to compute *β*, and unscaled standard errors of the coefficients:

$$\beta = \left(X^{T}X\right)^{-1}X^{T}Y,$$

$${uSE}_{\beta} = diag\left(X^{T}X\right).$$

Global coefficients *β* are sent back to the clients, which locally compute fitted log-CPM

$$\hat{Y}^{i} = X^{i}\beta,$$

and the noisy sums of squared errors

$$SSE^{i} = \sum\limits_{s=1}^{m^{i}} {\left(y^{i}_{s} - \hat{y}^{i}_{s}\right)^{2}} + N^{i}_{SSE},$$

where *s* is sample index and *m*^*i*^ is the total number of samples in the *i*th client.

The aggregator collects noisy *SSE*^*i*^ from clients, receives global noise

$$N_{SSE}=\sum\limits_{i}{N^{i}_{SSE}} $$

from the compensator, and computes estimated residual standard deviations for each gene:

$$\sigma = \sqrt{\frac{\sum\limits_{i}{SSE^{i}} - N_{SSE}}{\left(\sum\limits_{i}m^{i}\right)-k }} $$

The fifth step involves only *β*, *σ*^2^, and unscaled standard errors, and therefore does not require to be federated. All subsequent computations are performed on the side of the aggregator in the same way as done by the original *limma voom*.

### Meta-analysis approaches

Three classes of meta-analysis approaches can be distinguished: effect size combination methods, *p*-value combination methods, and non-parametric methods [33]. Effect size combination methods estimate variances of effect sizes for every gene and compute global effect sizes as a weighted sum of local effect sizes divided by the sum of all weights. This class includes the fixed effects model (FEM) and the random effects model (REM), which differ in the way they compute weights [30]. FEM calculates the weights as the inverse of the within-study variance. REM assumes that total variance includes within-study and between-study variance components and calculates the inverse of their sum. Both methods calculate *p*-values given global effect sizes and assuming their normal distribution. We chose REM since it is more robust to data heterogeneity than FEM and more widely used [83].

*P*-value combination methods are based on the assumption that the sum, minimum or maximum of log-transformed *p*-values obtained in independent studies follow a certain distribution [33]. These methods are thought to be more suitable for imbalanced scenarios than effect size combination methods [84]. From this class of methods, we chose Fisher’s method [27] because it is most sensitive to small *p*-values [85] and Stouffer’s method (also known as z-method) [28] since it was shown to be superior to Fisher’s method in some cases [86].

Non-parametric rank-based methods estimate global permutation-based *p*-value, by comparing the sum or the product of ranks obtained for the observed matrix of ranks with the same summary statistics calculated on shuffled rank matrices. Although the Rank Product method [29] is much more computationally expensive than the Rank Sum, the first gives more robust results [87].

In this work, we used the REM and Fisher’s method from *metaVolcanoR* package [88], the implementation of Stouffer’s method from *MetaDE* package [89] and *RankProd* package [90] for Rank Product method. For all selected meta-analysis methods except REM, global fold change was calculated as a mean of local fold changes.

### Evaluation

The main result of differential expression analysis is a list of genes with *p*-values and log-fold changes, reflecting the significance and the strength of differential expression, respectively. To validate the results of *Flimma* and demonstrate its advantage over meta-analysis approaches, we compared the *Flimma* and meta-analysis results obtained on artificial dataset splits to the results of *limma voom* applied on the aggregated datasets.

We chose two large datasets comprising RNA-seq gene expression profiles of human-derived samples. The first dataset included 850 expression profiles of human breast tumors from TCGA-BRCA cohort [55], classified as luminal or basal subtypes and annotated with patient age and tumor stage. We searched for genes differentially expressed between luminal and basal subtypes and included the age of diagnosis and tumor stage as covariates. The second dataset comprised 1277 skin expression profiles from GTEx [56] with sun exposure as target class label and patient age and sex as covariates. Each dataset has been divided into cohorts to model the multi-party setting under various scenarios (see the “Datasets” section for details).

In all tests, we applied *limma voom* on the complete dataset and on each of its partitions independently. The p-values and effect sizes computed by *limma voom* on the aggregated datasets were treated as ground truth, and those obtained on cohorts were used as input for the meta-analysis methods, which aggregated them to the global *p*-values.

To avoid manual execution of *Flimma* GUI for every test, we used a script performing exactly the same computations as the web version of *Flimma*. The code for running *Flimma* and its baselines, and the instructions for data download and preprocessing are available at GitHub (https://github.com/ozolotareva/flimma) [91] and at Zenodo (doi:10.5281/zenodo.5711972) [92] under the terms of the Apache 2.0 license. *Flimma* and the methodology of its evaluation are described in AIMe registry [93] at https://aime-registry.org/report/v6v9dj.

For each method, we considered a gene determined as differentially expressed, if it has |*log*(*FC*)|>1, and BH-adjusted *p*-value < 0.05. For the results produced by each method, we computed the RMSE, the precision, the recall, the F1 score, the Pearson, and the Spearman correlation. Since only a small number of the most significantly differentially expressed genes is of interest for some research tasks, we have also investigated how the performance of the methods varies with the numbers of top-ranked genes selected.

### Datasets

#### TCGA breast cancer data

Unprocessed read counts summarized to gene-level and clinical annotations of samples were downloaded from https://gdac.broadinstitute.org/runs/stddata__2016_01_28/data/BRCA/20160128. 850 expression profiles classified as luminal, or basal-like subtypes and annotated with the age of diagnosis and tumor stage were kept. Although breast cancer samples are classified into 4–6 subtypes [94–96], we focused on the most frequent subtypes for evaluation purposes. Luminal and basal subtypes are well distinguishable at the level of gene expression [55, 94] (Additional file 4: Figure S3A). We searched for genes differentially expressed between these subtypes and included the age of diagnosis and tumor stage as covariates. The luminal subtype is subdivided into luminal A (LumA) and luminal B (LumB) subtypes [95]. However, the LumA subtype was not included in the model as a covariate and we modeled the presence of an unknown disease subtype in our experiments.

#### GTEx skin data

Raw read counts per gene were obtained from the GTEx v8 portal website (https://www.gtexportal.org/home/datasets). Expression profiles of sun-exposed and non-sun-exposed skin samples annotated with mean ischemic time and sex were kept. The resulting dataset comprises 1277 expression profiles of 677 sun-exposed and 600 non-sun-exposed skin samples, also annotated with sex and ischemic time. In contrast to the TCGA-BRCA dataset, a smaller fraction of genes was differentially expressed between sun-exposed and non-exposed skin samples (Additional file 4: Figure S3B). Besides patient age and sex, samples were annotated with ischemic time, i.e. the time between patient death or sample withdrawal and sample fixation, or freezing. Ischemic time was not included in linear models but varied between cohorts in imbalanced scenarios, thus serving as an unknown confounder related to differences in sample preprocessing.

#### Generation of artificially distributed and heterogeneous datasets

To demonstrate the robustness of *Flimma*, we split both datasets differently in a balanced, a mildly imbalanced, and a strongly imbalanced scenario. In the balanced scenario, each sample was randomly assigned to one of three equal-sized cohorts with a similar distribution of covariates. In the imbalanced scenarios, the fractions of target classes and the distributions of some covariates differed among cohorts. Cohort sizes were unequal and related as 1:2:4 and 1:3:9 for the mildly and the strongly imbalanced scenarios, respectively. In the TCGA-BRCA dataset, we introduced an imbalance of luminal and basal subtype frequencies and, in addition, changed the frequency of the LumA subtype (Table 1). In the GTEx skin dataset, the fraction of sun-exposed skin samples and the median of mean ischemic times were made unequal between cohorts in imbalanced scenarios (Table 2).

#### GEO datasets

Raw read counts for three breast cancer cohorts from GSE129508 [57], GSE149276 [58], and GSE58135 [59] were obtained from ARCHS^4^ [60] (https://maayanlab.cloud/archs4/). ARCHS^4^ collected raw reads from publicly available human and mouse GEO datasets and uniformly preprocessed them. Raw reads from each human-derived sample were pseudo-aligned against the GRCh38 human reference genome and quantified by *kallisto* [97]. Since in our experiment we searched for genes differentially expressed between human breast cancer subtypes, we have chosen datasets comprising patient-derived breast tumor samples and excluded xenografts and cell lines. We also excluded samples annotated as cell lines from GSE58135 and post-intervention samples from GSE129508. Intrinsic breast cancer subtypes were predicted using the *genefu* R package [98]. Same as before, we searched for genes, differentially expressed between the luminal and basal subtypes. Luminal A subtype and the sequencing center were added to the model as covariates.

## Supplementary Information

**Additional file 1** Table S1.

**Additional file 2** Table S2

**Additional file 3** Table S3

**Additional file 4** Supplementary Text and Figures S1-S3

**Additional file 5** Table S4

**Additional file 6** Table S5

**Additional file 7** Table S6

**Additional file 8** Table S7

**Additional file 9** Review history.

## Data Availability

All data analyzed during this study were obtained from publicly available resources. Gene expressions of breast tumors from TCGA-BRCA dataset [55] were obtained from Broad GDAC Firehose (https://gdac.broadinstitute.org/runs/stddata__2016_01_28/data/BRCA/20160128/). Uniformly preprocessed gene-level read counts for three additional independently generated breast cancer datasets GSE129508 [57], GSE149276 [58], and GSE58135 [59] were downloaded from ARCHS^4^ [60] (https://maayanlab.cloud/archs4/). Gene expression of sun-exposed and not sun-exposed skin were downloaded from GTEx [56] website (https://gtexportal.org/home/datasets). The code for running *Flimma* and its baselines, and the instructions for data download and preprocessing are available at GitHub (https://github.com/ozolotareva/flimma) [91] and Zenodo (https://zenodo.org/record/5711972#.YZrXm5HMIrM) [92].
