# Supplementary material for: Flimma: a federated and privacy-aware tool for differential gene expression analysis
Source: Genome Biol. 2021 Dec 14;22:338. doi: 10.1186/s13059-021-02553-2 (PMC8670124; doi:10.1186/s13059-021-02553-2)
Supplement: Supplementary file 4 — Additional file 4 Supplementary Text and Figures S1-S3 [file 13059_2021_2553_MOESM4_ESM.pdf]

Additional file 4

Supplementary Text

Alternatively to the approach used by *Flimma*, cohort batch effects could be adjusted for based on one or several first principal components. However, our results suggest that PCA-based batch effect correction with one to four principal components seems to be not effective for gene expression data (Additional file 8: Table S7).

To additionally validate the lists of differentially expressed genes produced by *Flimma* and *limma voom* with PCA-based correction on three GEO datasets, we compared them with the list obtained on TCGA-BRCA in centralized analysis. Since TCGA-BRCA is much larger than the GEO dataset (850 samples vs. 132) and seems to be unaffected by center-specific batch effects (Figure 4), we assumed that the signature discovered in this dataset by *limma voom* is reliable and, thus, used it as the second comparison partner. Again, on GEO datasets, the list of differentially expressed genes obtained by *Flimma* was more similar to the TCGA-BRCA gene list than lists produced by *limma voom* with 1-4 first principle components taken as covariates. This validates the reliability of *Flimma* regarding its ability to replicate the result obtained on a larger and more heterogeneous cohort (Additional file 8: Table S7).

Supplementary Figures

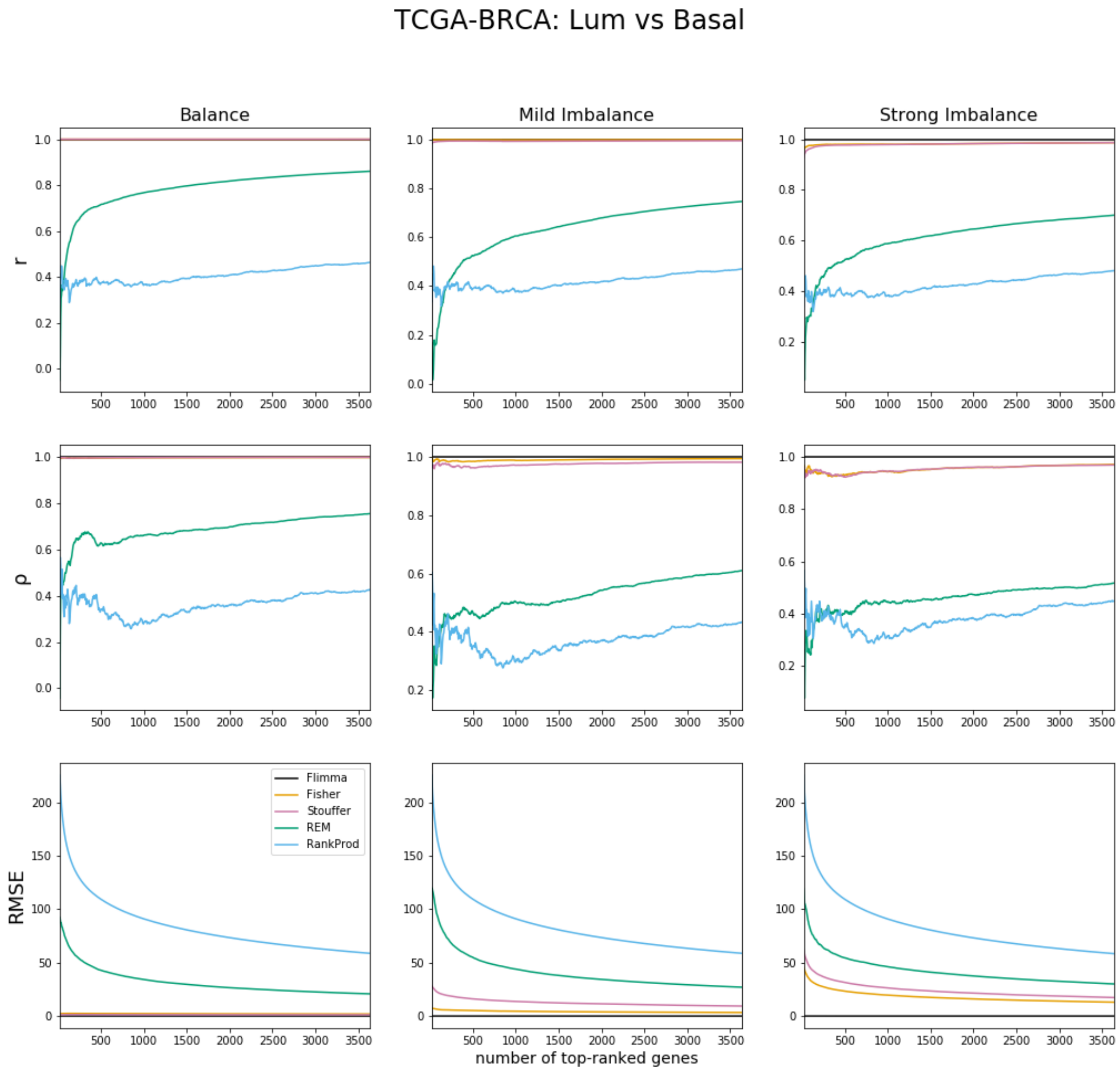

Figure S1. The dependency of Pearson's and Spearman's correlation coefficients and RMSE on the number of top-ranked genes considered to be differentially expressed in TCGA-BRCA data.

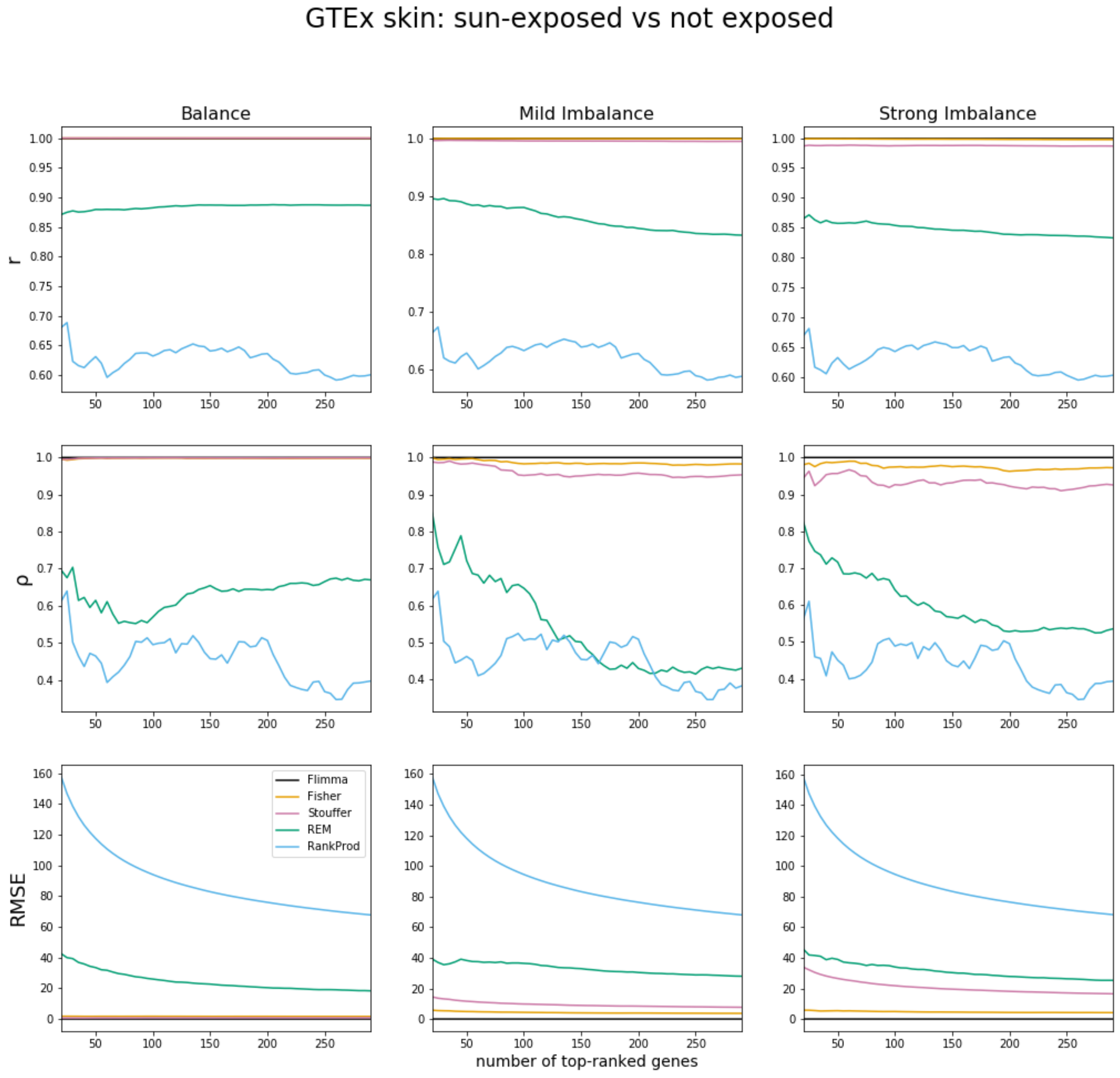

Figure S2. The dependency of Pearson's and Spearman's correlation coefficients and RMSE on the number of top-ranked genes considered to be differentially expressed in GTEx skin dataset.

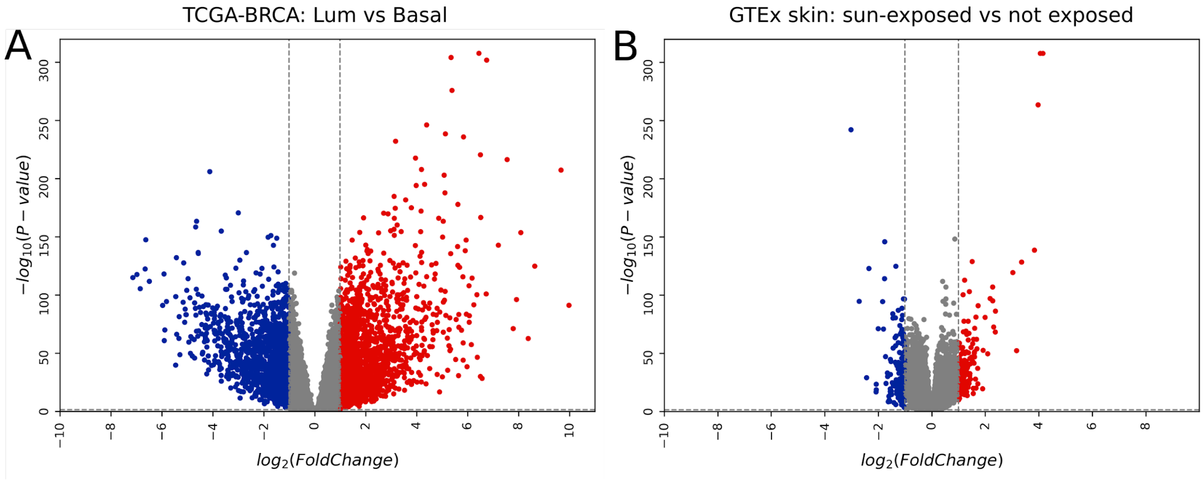

Figure S3. The results of differential expression analysis performed by *limma-voom* on the aggregated TCGA-BRCA (A) and GTEx Skin datasets (B). 3635 and 288 genes were differentially expressed with absolute log-fold change |logFC| > 1 and BH-adjusted p-value < 0.05 according to *limma voom* applied on pooled TCGA-BRCA and GTEx Skin datasets respectively.
