# Supplementary material for: Flimma: a federated and privacy-aware tool for differential gene expression analysis
Source: Genome Biol. 2021 Dec 14;22:338. doi: 10.1186/s13059-021-02553-2 (PMC8670124; doi:10.1186/s13059-021-02553-2)
Supplement: Supplementary file 9 — Additional file 9 Review history. [file 13059_2021_2553_MOESM9_ESM.docx]

Review History

**First round of review**

**Reviewer 1**

**Were you able to assess all statistics in the manuscript, including the appropriateness of statistical tests used?**

Yes, and I have assessed the statistics in my report.

**Were you able to directly test the methods?**

Yes

**Comments to author:**

The paper introduces a tool called flimma, which essentially is a distributed reconception and implementation of the famous gene expression data analysis tool limma voom. The identification of differentially expressed genes (DEGs) often is the first basic step in biomarker profiling and biomedical/clinical data analysis. The problem inherent particularly in RNAseq data is that the raw data may (as it is sequencing data) may reveal the ID and (genetic) traits of individuals. Hence, it may not simply be exchanged between participating sites for a centralized analysis with tools like limma, DeSeq2 or EdgeR. The authors introduce a federated version of limma which seems to provide the same results, e.g., p-values etc. as limma voom but without the necessity to share the raw data. The authors compared flimma with limma (on centralized data) but also on artificially split/pseudo-federated data in different scenarios of having imbalanced confounder distributions and data set sizes.

The web site provides exhaustive instructions and a demo video on how to use it. Thus, flimma may have the potential to become the standard tool for clinical gene expression data analysis (at least for DEG

identification) for multi-center studies.

The motivation RNAseq data reveals genetic traits and missing clinical data could be more elaborated. At the moment it's just two or three sentences with a lot of citations. A bit more explanation giving the reader a better idea of why and how privacy can be violated when using centralized DEG profiling would be good.

While the authors make a reasonable effort in comparing against meta-analysis tools, other privacy-enhancing technologies (PETs, e.g. differential privacy, secure multi-party computation, homomorphic encryption) are not mentioned. Why federated computing and not any of the other technologies? I can imagine that this is what the authors tested first and it seemed to work wrt. accuracy (same as limma) and run time. If this is the case, the authors should state this clearly (i.e. that other PET approaches might also work but have not been tested). Do no other PET tools for DEG identification not exist? If not, this should be stated clearly as well - maybe together with one sentence for each PET in the outlook if they could work as well (maybe even better?).

How about other omics data (proteomics, metabolomics, microbiomics) where limma could potentially be applied? Worth checking/trying? Future work? Not enough data to evaluated? Please discuss this at least in the discussion, see e.g., pubmed:25821719, pubmed:26859769, or https://bioconductor.org/packages/release/bioc/vignettes/yamss/inst/doc/yamss.html

The authors did not mention potentially unknown confounders factors (arising e.g. from population structure, as in GWAS). Does this not matter here, i.e. for gene expression data? If it matters, maybe a

federated PCA is required to account for this? That may need a short discussion in the paper or supplement, if it is relevant. Here two papers with potentially relevant background:

pubmed:26797895, pubmed:19777146

An effective batch effect correction would indeed be desirable, as clearly identified as shortcoming by the authors. Which method would be most feasible / straightforward to be integrated as federated version into a future flimma package and why?

There is no license for the software mentioned. Is it GPL3? Should be stated in the paper.

**Reviewer 2**

**Were you able to assess all statistics in the manuscript, including the appropriateness of statistical tests used?**

Yes, and I have assessed the statistics in my report.

**Were you able to directly test the methods?**

No

**Comments to author:**

In this manuscript, Zolotareva et al. introduce a web service called filmma for federated differential gene expression analysis. The software allows multiple clients, each with their own gene expression dataset, to perform a different gene expression analysis jointly over all of the datasets without having to share the raw data among the clients. The analysis pipeline is closely modeled after the existing limma voom pipeline. The authors show that their approach produces the same results as limma voom in the federated setting, whereas existing meta analysis approaches suffer from inaccurate results when the given data distribution is highly unbalanced between different clients. The data sharing problem addressed by the authors is timely. The federation technique used by the authors by itself is relatively straightforward and draws from prior literature, which involves pooling aggregate statistics across parties to compute the linear regression coefficients. The experimental results clearly show that flimma is superior to meta-analysis in terms of accuracy, which is expected as flimma replicates the functionality of the joint analysis based on limma voom. I have two major concerns about this work.

The authors claim that flimma is privacy-preserving, however this claim appears to be based on the misleading belief that not sharing the raw data automatically implies that the data is safe. This is not necessarily the case, because information about the underlying data can still be leaked through aggregate-level information, such as the intermediate terms in the flimma workflow (X'X, X'Y) that are openly shared with the central server under the assumption it is safe to do so. However, if the design matrix contains two columns that differ only by a single position for example, then one can in fact extract the complete gene expression profile of an individual by subtracting the corresponding rows of the X'Y terms (concatenated across all genes). This type of reconstruction attack can pose a real threat when the attacker has some side information about the private dataset that allows them to linearly combine the covariate features to single out a specific individual in the cohort. This is just one possible leakage in flimma; there may be others. Privacy claims that are not based on firm grounds can have severe consequences in practice as they give a false sense of security to the potential users. I would strongly suggest that the authors carefully analyze and address potential vulnerabilities in their proposed workflow before deploying the service in the real world.

All of the experiments presented in the manuscript consider a distributed setup that is simulated by randomly partitioning a benchmark dataset into different subsets. One of the key hurdles in federated analysis is the lack of data harmonization and experimental batch effects across different labs. The authors need to demonstrate that flimma is robust under these circumstances by testing it on a collection of gene expression datasets generated in different labs with potential batch effects, given that this is the target use case of flimma. It would be important for flimma to provide tools to correct for these differences, also in a federated and privacy-preserving manner. Given public data repositories like ArrayExpress, I do not expect data acquisition to be a major hurdle for incorporating this addition to the work.

The website hosted by the authors is well organized and provides user friendly instructions. However, the provided client software did not run on my machine.

**Authors Response**

**Point-by-point responses to the reviewers’ comments:**

Reviewer #1

The paper introduces a tool called flimma, which essentially is a distributed reconception and implementation of the famous gene expression data analysis tool limma voom. The identification of differentially expressed genes (DEGs) often is the first basic step in biomarker profiling and biomedical/clinical data analysis. The problem inherent particularly in RNAseq data is that the raw data may (as it is sequencing data) may reveal the ID and (genetic) traits of individuals. Hence, it may not simply be exchanged between participating sites for a centralized analysis with tools like limma, DeSeq2 or EdgeR. The authors introduce a federated version of limma which seems to provide the same results, e.g., p-values etc. as limma voom but without the necessity to share the raw data. The authors compared flimma with limma (on centralized data) but also on artificially split/pseudo-federated data in different scenarios of having imbalanced confounder distributions and data set sizes.

The web site provides exhaustive instructions and a demo video on how to use it. Thus, flimma may have the potential to become the standard tool for clinical gene expression data analysis (at least for DEG identification) for multi-center studies.

The motivation RNAseq data reveals genetic traits and missing clinical data could be more elaborated. At the moment it's just two or three sentences with a lot of citations. A bit more explanation giving the reader a better idea of why and how privacy can be violated when using centralized DEG profiling would be good.

*We extended the paragraph:*

*Schadt et al. have shown that genotypes can be inferred from expression levels of eQTL-controlled genes and sensitive information — for instance medical history, phenotypic traits, and family relationships can be revealed (Schadt, Woo, and Hao 2012). Matching predicted genotypes to known ones allowed for identifying individuals with an accuracy of up to 99% in optimal settings, i.e. when the microarray platform, tissue type, and ancestry were the same for expression and eQTL datasets. Harmanci and Gerstein proposed a measure of individual-characterizing information leakage and investigated its dependence on genotype predictability given the expression dataset (Harmanci and Gerstein 2016). They developed a framework to assess privacy risks before publishing the data. They have also presented a simplified but effective attack scheme where homozygous genotypes were predicted from extreme gene expression values.*

While the authors make a reasonable effort in comparing against meta-analysis tools, other privacy-enhancing technologies (PETs, e.g. differential privacy, secure multi-party computation, homomorphic encryption) are not mentioned. Why federated computing and not any of the other technologies? I can imagine that this is what the authors tested first and it seemed to work wrt. accuracy (same as limma) and run time. If this is the case, the authors should state this clearly (i.e. that other PET approaches might also work but have not been tested). Do no other PET tools for DEG identification not exist? If not, this should be stated clearly as well - maybe together with one sentence for each PET in the outlook if they could work as well (maybe even better?).

*As recommended, we added a paragraph about PETs to Introduction:*

*There are several other privacy-enhancing techniques alternative to federated learning such as differential privacy (Dwork et al. 2006) (DP), homomorphic encryption (Gentry 2009) (HE), and secure multi-party computation (Cramer, Damgård, and Nielsen 2015) (SMPC) which are used to analyze biomedical data (Torkzadehmahani et al. 2020). DP perturbs the data or results by adding noise to them. Although it is complementary to federated learning, it might dramatically impact the accuracy of the results. HE performs computation on the encrypted data from the participants. It suffers from two practical disadvantages (Chialva and Dooms 2018): it supports a limited number of operations such as addition and multiplication, and consequently, it requires approximations to compute non-linear operations (e.g. computing the inverse of covariance matrix in gene expression analysis), leading to accuracy loss in the final results. More importantly, it is computationally expensive and does not scale linearly for large distributed biomedical datasets. In SMPC, each participant computes secret shares from the data and shares them with the computing parties. The computing parties calculate the intermediate results and exchange them among each other to compute the final results. Similar to HE, SMPC is not scalable to heavily distributed datasets because a huge amount of secret shares from the participants have to be processed by a few computing parties. Hence, federated learning is a suitable alternative balancing performance and scalability.*

How about other omics data (proteomics, metabolomics, microbiomics) where limma could potentially be applied? Worth checking/trying? Future work? Not enough data to evaluated? Please discuss this at least in the discussion, see e.g., pubmed:25821719, pubmed:26859769, or https://bioconductor.org/packages/release/bioc/vignettes/yamss/inst/doc/yamss.html

*We agree that the development of Flimma modifications suitable for the analysis of other omics data types is a prospective direction of future work. We added one more paragraph to Discussion:*

*Although limma has been initially developed for differential gene expression analysis, it is widely used for the analysis of various omics data types, e.g. proteomics (Kammers et al. 2015; Zhu et al. 2020), metabolomics (Myint et al. 2017), and microbiomics (Zhang et al. 2019). Therefore, we plan the development of Flimma modifications suitable for the analysis of other omics data types in the future.*

The authors did not mention potentially unknown confounders factors (arising e.g. from population structure, as in GWAS). Does this not matter here, i.e. for gene expression data?

If it matters, maybe a federated PCA is required to account for this? That may need a short discussion in the paper or supplement, if it is relevant. Here two papers with potentially relevant background: pubmed:26797895, pubmed:19777146

*Gene expression data are affected by multiple known and unknown factors, including genetic heterogeneity of the population. However, unlike genotype data, the impact of the latter on the total variation is negligible compared to the impact of technical factors, related to sample preparation and expression profiling in the lab. Brown et al. have shown that even after the adjustment for experimental batches, populational structure remains invisible on conventional PCA even after correction for technical batches (Brown, Bray, and Pachter 2018). They found that correlation becomes evident when a canonical correlation analysis is performed on first components from PCA-projections of genotype and expression matrices. This approach may be adopted for correction for unknown population structure but it would require genotype data for the same patients which is not always available. The question whether this method would be superior to simple modeling of population genetic structure with additional variables (as in Flimma and e.g. in pubmed:26797895) is the subject of a separate study. We added a short experiment demonstrating that the approach used by Flimma gives better results than PCA-based batch effect correction (Supplementary text and Table S7).*

*Nevertheless, we agree that federated PCA is useful for quality control checks and we consider adding it to Flimma in later versions. Currently, a stand-alone app for federated PCA for FeatureCloud system (https://featurecloud.eu/) is under development since it is a key step for analysis of GWAS data.*

An effective batch effect correction would indeed be desirable, as clearly identified as shortcoming by the authors. Which method would be most feasible / straightforward to be integrated as federated version into a future flimma package and why?

*Indeed, gene expression levels of technical replicates measured in two laboratories may drastically differ since their measurements are affected by multiple experimental factors. Following the recommendations of limma author (https://support.bioconductor.org/p/123048/) and (Nygaard, Rødland, and Hovig 2016), the current version of Flimma effectively addresses this issue by adding covariates, such as those that account for the effect of clinical center, to the model.*

*An alternative approach would be to remove batch-effects from the data before differential expression analysis. However, not all batch-effect correction methods are compatible with the limma voom method which involves count-based statistics. We think that ComBat-Seq would be the best choice for our setting when each center represents a technical batch. RUVSeq removes variation emerged from unknown technical sources and does not require to specify technical batches. It requires either control genes with stable expression whose existence is not guaranteed, or replicated samples measured at different centers, which may not be available due to data protection. We mentioned ComBat-Seq and RUVSeq methods in the discussion:*

*Another prospective direction for future work is the development of accessory tools for gene expression analysis. This includes for example, federated Principle Component Analysis (PCA), useful for quality control, or federated batch effect correction methods, such as ComBat or RUVSeq (Risso et al. 2014). Although we have shown that the current version of Flimma effectively handles batch effects, other analyses of expression data such as clustering or classification might require transformed data.*

*We added an analysis demonstrating that the approach used by Flimma allows for adjusting for center-specific batch effects and seems to be more successful than PCA-based approach (see Supplementary text and Table S7). We applied Flimma on three additional breast cancer datasets and demonstrated that the resulting gene list is 1) highly similar to the result limma voom applied on ComBat-seq corrected data, 2) resembles the result obtained on TCGA-BRCA cohort in centralized analysis.*

There is no license for the software mentioned. Is it GPL3? Should be stated in the paper.

*The Flimma source code is distributed under MIT. We mentioned it in the text and will make the repository public when the paper is published.*

Reviewer #2

In this manuscript, Zolotareva et al. introduce a web service called filmma for federated differential gene expression analysis. The software allows multiple clients, each with their own gene expression dataset, to perform a different gene expression analysis jointly over all of the datasets without having to share the raw data among the clients. The analysis pipeline is closely modeled after the existing limma voom pipeline. The authors show that their approach produces the same results as limma voom in the federated setting, whereas existing meta analysis approaches suffer from inaccurate results when the given data distribution is highly unbalanced between different clients. The data sharing problem addressed by the authors is timely. The federation technique used by the authors by itself is relatively straightforward and draws from prior literature, which involves pooling aggregate statistics across parties to compute the linear regression coefficients. The experimental results clearly show that flimma is superior to meta-analysis in terms of accuracy, which is expected as flimma replicates the functionality of the joint analysis based on limma voom. I have two major concerns about this work.

The authors claim that flimma is privacy-preserving, however this claim appears to be based on the misleading belief that not sharing the raw data automatically implies that the data is safe. This is not necessarily the case, because information about the underlying data can still be leaked through aggregate-level information, such as the intermediate terms in the flimma workflow (X'X, X'Y) that are openly shared with the central server under the assumption it is safe to do so. However, if the design matrix contains two columns that differ only by a single position for example, then one can in fact extract the complete gene expression profile of an individual by subtracting the corresponding rows of the X'Y terms (concatenated across all genes). This type of reconstruction attack can pose a real threat when the attacker has some side information about the private dataset that allows them to linearly combine the covariate features to single out a specific individual in the cohort. This is just one possible leakage in flimma; there may be others. Privacy claims that are not based on firm grounds can have severe consequences in practice as they give a false sense of security to the potential users. I would strongly suggest that the authors carefully analyze and address potential vulnerabilities in their proposed workflow before deploying the service in the real world.

*We agree with the reviewer that exchange of intermediate results may lead to potential information leak in particular cases, and private data may be reconstructed from and in some cases. Besides the case described by the reviewer, there are some other potential vulnerabilities we described in a new subsection “Data security” added to Methods. To protect the users from these reconstruction attacks, we modified the client app such that it does not accept inputs not passing security checks. Furthermore, the model parameters are only shared with the server over an encrypted communication using HTTPs. Therefore, the other clients or an outsider will not have direct access to the model parameters of a particular client.*

All of the experiments presented in the manuscript consider a distributed setup that is simulated by randomly partitioning a benchmark dataset into different subsets. One of the key hurdles in federated analysis is the lack of data harmonization and experimental batch effects across different labs. The authors need to demonstrate that flimma is robust under these circumstances by testing it on a collection of gene expression datasets generated in different labs with potential batch effects, given that this is the target use case of flimma. It would be important for flimma to provide tools to correct for these differences, also in a federated and privacy-preserving manner. Given public data repositories like ArrayExpress, I do not expect data acquisition to be a major hurdle for incorporating this addition to the work.

*We agree that the presence of strong experimental batch effects in the data is one the main pitfalls of gene expression analysis. To show that Flimma can handle experimental batch effects we added a new analysis to the paper (see subsection Robustness to batch effects). We applied Flimma on three more independent breast cancer datasets and demonstrated that the resulting gene list is very similar (Spearman correlation 0.96) to the gene list obtained by limma voom on these datasets after the removal of cohort-specific batch effect by ComBat-Seq.*

The website hosted by the authors is well organized and provides user friendly instructions. However, the provided client software did not run on my machine.

*The client package has been tested on Windows 10, Ubuntu 18.04, and Mac OS X 10. If you use a different system we suggest following the instructions in the GitHub README and running the development version of the client package.*

*References*

*Brown, Brielin C., Nicolas L. Bray, and Lior Pachter. 2018. “Expression Reflects Population Structure.” PLoS Genetics 14 (12): e1007841.*

*Chialva,Diego, and Dooms,Ann. Conditionals in Homomorphic Encryption, and Machine Learning Applications Diego Chialva and Ann Dooms. 2018. arXiv [cs.CR]. arXiv. https://arxiv.org/pdf/1810.12380.pdf.*

*Cramer, Ronald, Ivan Bjerre Damgård, and Jesper Buus Nielsen. 2015. Secure Multiparty Computation. Cambridge University Press.*

*Dwork, Cynthia, Frank McSherry, Kobbi Nissim, and Adam Smith. 2006. “Calibrating Noise to Sensitivity in Private Data Analysis.” Theory of Cryptography. https://doi.org/10.1007/11681878_14.*

*Gentry, Craig. 2009. A Fully Homomorphic Encryption Scheme.*

*Harmanci, Arif, and Mark Gerstein. 2016. “Quantification of Private Information Leakage from Phenotype-Genotype Data: Linking Attacks.” Nature Methods 13 (3): 251–56.*

*Kammers, Kai, Robert N. Cole, Calvin Tiengwe, and Ingo Ruczinski. 2015. “Detecting Significant Changes in Protein Abundance.” EuPA Open Proteomics 7 (June): 11–19.*

*Myint, Leslie, Andre Kleensang, Liang Zhao, Thomas Hartung, and Kasper D. Hansen. 2017. “Joint Bounding of Peaks Across Samples Improves Differential Analysis in Mass Spectrometry-Based Metabolomics.” Analytical Chemistry 89 (6): 3517–23.*

*Nygaard, Vegard, Einar Andreas Rødland, and Eivind Hovig. 2016. “Methods That Remove Batch Effects While Retaining Group Differences May Lead to Exaggerated Confidence in Downstream Analyses.” Biostatistics 17 (1): 29–39.*

*Risso, Davide, John Ngai, Terence P. Speed, and Sandrine Dudoit. 2014. “Normalization of RNA-Seq Data Using Factor Analysis of Control Genes or Samples.” Nature Biotechnology. https://doi.org/10.1038/nbt.2931.*

*Schadt, Eric E., Sangsoon Woo, and Ke Hao. 2012. “Bayesian Method to Predict Individual SNP Genotypes from Gene Expression Data.” Nature Genetics 44 (5): 603–8.*

*Torkzadehmahani, Reihaneh, Reza Nasirigerdeh, David B. Blumenthal, Tim Kacprowski, Markus List, Julian Matschinske, Julian Späth, et al. 2020. “Privacy-Preserving Artificial Intelligence Techniques in Biomedicine.” arXiv [cs.CR]. arXiv. http://arxiv.org/abs/2007.11621.*

*Zhang, Xiang, Max Nieuwdorp, Albert K. Groen, and Aeiko H. Zwinderman. 2019. “Statistical Evaluation of Diet-Microbe Associations.” BMC Microbiology 19 (1): 90.*

*Zhu, Yafeng, Lukas M. Orre, Yan Zhou Tran, Georgios Mermelekas, Henrik J. Johansson, Alina Malyutina, Simon Anders, and Janne Lehtiö. 2020. “DEqMS: A Method for Accurate Variance Estimation in Differential Protein Expression Analysis.” Molecular & Cellular Proteomics: MCP 19 (6): 1047–57.*

**Second round of review**

**Reviewer 2**

The additional results demonstrating batch effect correction using Flimma is informative and helpful. However, the authors have only partially addressed my comment regarding the lack of privacy guarantee of the proposed workflow.

A new paragraph has been added to Methods section describing a number of potential attack scenarios. Yet the main text does not have any mention of this caveat. Given that privacy protection is the main motivation of Flimma, why isn't the possibility that individual-level data could still be leaked more centrally discussed in the manuscript?

The proposed mitigation strategy is to disallow inputs that appear vulnerable according to a small set of pre-defined rules. I am not convinced this provides meaningful protection, since minor modifications to the attacks can still leak data while evading the proposed mitigation strategy. For example, it is stated that if the design matrix X contains an identity submatrix of size m, then the gene expression profiles could be extracted. Flimma now detects this case and rejects such an input, but what about identity submatrices of size less than m? Those can still reveal individual-level data, albeit for a smaller subset of samples. What about submatrices that are not identity matrices but are invertible, which can also leak data? I am concerned that manually enumerating all of these possibilities and excluding them may limit the applicability of Flimma. The authors need to better justify that the proposed mitigation is sufficient in practice.

Minor comments:

The statement that HE and SMPC do not scale to large biomedical datasets need to be better qualified. For certain applications these methods can lead to efficient protocols. E.g. see recent works on GWAS such as [1] and [2]. Another important distinction to make is that HE and SMPC are cryptographic methods with formally defined security guarantees unlike federated learning.

A small typo: "HTTPs" -> "HTTPS"

[1] https://doi.org/10.1073/pnas.1918257117

[2] http://dx.doi.org/10.1038/nbt.4108

**Authors Response**

**Point-by-point responses to the reviewers’ comments:**

Reviewer #2

The additional results demonstrating batch effect correction using Flimma is informative and helpful. However, the authors have only partially addressed my comment regarding the lack of privacy guarantee of the proposed workflow.

A new paragraph has been added to Methods section describing a number of potential attack scenarios. Yet the main text does not have any mention of this caveat. Given that privacy protection is the main motivation of Flimma, why isn't the possibility that individual-level data could still be leaked more centrally discussed in the manuscript?

*Response:*

*We mentioned this potential vulnerability in the discussion.*

To enhance data privacy, Flimma uses a hybrid federated approach where parameters potentially susceptible to the reconstruction attack are protected by adding random noise, which is further compensated by an additional computing party to preserve the accuracy of the results. This security feature minimizes the vulnerability to reconstruction attacks, although does not fully exclude it. However, in reality, the risk of a successful reconstruction attack is negligible, because it would require compromising two servers located at distant physical sites. Therefore, Flimma assumes honest-but-curious (semi-honest) parties, which stick with the protocol and follow it but they try to reconstruct the data from the model parameters. Moreover, the assumption is that the collusion does not happen, i.e. the server or compensator never exchange the individual noisy parameters or noise values from the clients with each other, and there are at least two clients participating in the study.

*The explanations of hybrid approach are provided below and in the updated version of the paper.*

The proposed mitigation strategy is to disallow inputs that appear vulnerable according to a small set of pre-defined rules. I am not convinced this provides meaningful protection, since minor modifications to the attacks can still leak data while evading the proposed mitigation strategy. For example, it is stated that if the design matrix X contains an identity submatrix of size m, then the gene expression profiles could be extracted. Flimma now detects this case and rejects such an input, but what about identity submatrices of size less than m? Those can still reveal individual-level data, albeit for a smaller subset of samples. What about submatrices that are not identity matrices but are invertible, which can also leak data?

I am concerned that manually enumerating all of these possibilities and excluding them may limit the applicability of Flimma. The authors need to better justify that the proposed mitigation is sufficient in practice.

*Response:*

*After careful reconsideration, we fully agree that these mitigation strategies are not sufficient to protect against reconstruction attacks because it is impossible to oversee all scenarios where reconstruction can happen at the time of deployment. Therefore, we changed the implementation of Flimma such that clients do not share with any party unprotected local parameters. To achieve this, we have reimplemented parts of Flimma based on the hybrid federated framework HyFed (https://github.com/tum-aimed/hyfed), which uses SMPC to add noise to the local parameters. This hides the true parameters from the server while preserving the utility of the global model. The HyFed framework requires two remote servers, aggregator and compensator, physically available at two different locations. Now, before sharing parameters with the aggregator, each client adds noise to its local parameters, making the reconstruction of private data impossible. In turn, the noise generated by each client is sent to a separate server called compensator, summarizing local noise and sharing it with the aggregator. The aggregator summarizes noisy local parameters to global and subtracts global noise obtained from the compensator. In the new implementation, neither the aggregator nor the compensator have access to local parameters. This gives a much better privacy guarantee because a reconstruction attack would require compromising two servers in this case. Therefore, security checks previously described in the Data security section are not included in the current Flimma implementation. We also modified the Introduction, Fig. 1 and Implementation sections accordingly and removed the paragraph describing potential attack scenarios.*

Minor comments:

The statement that HE and SMPC do not scale to large biomedical datasets need to be better qualified. For certain applications these methods can lead to efficient protocols. E.g. see recent works on GWAS such as [1] and [2]. Another important distinction to make is that HE and SMPC are cryptographic methods with formally defined security guarantees unlike federated learning.

[1] https://doi.org/10.1073/pnas.1918257117

[2] http://dx.doi.org/10.1038/nbt.4108

*Response:*

*Thank you very much for bringing these works into the discussion, we mentioned them in the introduction. We removed from the Introduction all statements that HE and SMPC-based approaches do not scale linearly and wrote that pure federated learning does not guarantee perfect privacy, when SMPC and HE do. Now, instead of pure federated learning, we use a hybrid approach with an SMPC-like component (described above), which retains the advantages of our federated learning strategy with the privacy guarantees through SMPC.*

A small typo: "HTTPs" -> "HTTPS"

*Response:*

*Thank you for pointing out this mistake. The paragraph containing it was removed from the text because we changed the strategy and do not perform the described security checks in the current implementation.*

**Third round of review**

**Reviewer 2**

The authors responded to the remaining concerns about leakage in the shared parameters by reimplementing Flimma now based on HyFed--a new federated learning technique proposed by the authors in a recent preprint. The key idea behind HyFed is to hide the local updates by adding noise, aggregating the noisy updates across parties, then exactly revealing only the aggregated update by subtracting out the sum of noises. This is achieved by introducing a third party that collects all of the noises from each participant and share the aggregated mask to the central coordinator for revealing the global update. The core technique of HyFed unfortunately seems to lack novelty, given that the seminal paper from 2017 by Bonawitz et al. (doi: 10.1145/3133956.3133982) laid out the techniques for secure aggregation of local updates (precisely the goal of the HyFed) using standard cryptographic techniques. In fact the work of Bonawitz et al. provides additional features that HyFed does not support, including robustness to failure and security against malicious parties. The authors need to better justify their use of HyFed compared to existing techniques.

Despite the addition of HyFed, the manuscript still lacks a thorough treatment of privacy properties of the proposed system. HyFed hides local updates but still reveals global updates to the coordinator. The same type of leakage discussed in prior communication (which the authors acknowledged) applies to these global updates. It helps that now the reconstruction risk applies only to the combined cohort as opposed to individual sites’ data, but this possibility is not addressed in the manuscript. Existing federated learning solutions typically adopt differential privacy for addressing these leakage concerns, which is not considered. There is also the exceptional case where, with only two parties collaborating, revealing the global update is akin to revealing the local update since each party can guess the other party’s update based on the knowledge of their own. Lastly, the authors describe in the introduction that “only highly noisy model parameters are shared with the server”. This only describes what each site shares with the aggregator; each site also shares the used noise with the compensator. This is akin to uploading encrypted data to one party and uploading the decryption key to another party. These remaining considerations need to be clarified in the manuscript.

From a practical perspective, HyFed increases the complexity of the system by requiring two independent third parties to play the roles of compensator and aggregator. If these two servers are operated by the same entity, the assumption that the data won’t be combined between these two servers to leak local updates likely won’t be reassuring enough for the users. More detailed explanation of who can play these roles in practice would be helpful.

**Authors Response**

**Point-by-point responses to the reviewers’ comments:**

The authors responded to the remaining concerns about leakage in the shared parameters by reimplementing Flimma now based on HyFed--a new federated learning technique proposed by the authors in a recent preprint. The key idea behind HyFed is to hide the local updates by adding noise, aggregating the noisy updates across parties, then exactly revealing only the aggregated update by subtracting out the sum of noises. This is achieved by introducing a third party that collects all of the noises from each participant and share the aggregated mask to the central coordinator for revealing the global update. The core technique of HyFed unfortunately seems to lack novelty, given that the seminal paper from 2017 by Bonawitz et al. (doi: 10.1145/3133956.3133982) laid out the techniques for secure aggregation of local updates (precisely the goal of the HyFed) using standard cryptographic techniques. In fact the work of Bonawitz et al. provides additional features that

HyFed does not support, including robustness to failure and security against malicious parties. The authors need to better justify their use of HyFed compared to existing techniques.

*Indeed, HyFed relies on the idea of secret sharing proposed by Shamir 1979 (10.1145/359168.359176). Bonawitz et al. (doi: 10.1145/3133956.3133982) also use the secret sharing scheme to hide the local parameters from the other parties. Although Bonawitz et al. claimed that they implemented a prototype in java, they have NOT made their code publicly available. Moreover, it still suffers from the same limitation as HyFed, i.e. disclosing the global parameters to the server and other clients. Finally, it has been designed for mobile devices, where failures or network connection issues of clients is normal and provides mechanisms to cope with those situations at the cost of the system complexity. HyFed is an open-source framework and designed for multi-institutional collaborative settings, in which network connections are stable and client failures are rare. It supports federated mode of operation, where multiple clients can communicate with the server over the Internet through a secure communication using the HTTPS protocol. HyFed is based on the additive secret sharing scheme whose privacy regarding hiding the local parameters of the clients from the third parties has already been proven in the corresponding studies. HyFed is a less complex framework compared to Bonawitz et al. (doi: 10.1145/3133956.3133982), and using the minimum computing parties (i.e. server and compensator) makes it more scalable and suitable for practical applications although it is less robust against colluding parties.*

Despite the addition of HyFed, the manuscript still lacks a thorough treatment of privacy properties of the proposed system. HyFed hides local updates but still reveals global updates to the coordinator. The same type of leakage discussed in prior communication (which the authors acknowledged) applies to these global updates. It helps that now the reconstruction risk applies only to the combined cohort as opposed to individual sites’ data, but this possibility is not addressed in the manuscript.

*As we write in the Discussion, Flimma relies on a hybrid FL-SMPC scheme and does not guarantee perfect privacy but significantly enhances privacy. We assume that all computing parties are honest-but-curious and there is no collusion between any of them. In the proposed approach, local intermediate results are hidden from servers and other clients, but global intermediate results are revealed to the aggregator. Given that, Flimma does not eliminate all privacy risks and we discussed some of them in the Discussion section. An important consideration here is that a similar discussion is also applicable to meta-analysis approaches because they also reveal local statistics to third parties without providing any guarantee that these revealed statistics do not lead to privacy leakage.*

*We decided to exclude the detailed discussion of the potential attack from the paper text since we are not going to prove the perfect privacy of Flimma in this paper. Instead, we warn the user about the possibility of such attacks on the example of two potential attack scenarios (see Discussion) and leave privacy-preserving differential expression analysis as an open problem. Despite this, we strongly believe that Flimma is an important step towards secure differential gene expression analysis since its only privacy-aware alternative (meta-analyses) is not robust to data imbalance.*

*Additionally, in this letter of response, we would like to discuss the attack scenarios mentioned during the peer review process. We think that these scenarios are unlikely to happen in practice because they either*

*-	will not appear in practice because of data preprocessing,*

*-	fulfill when the inputs have properties making them useless for differential expression*

*analysis.*

- *Identity submatrix in the expression matrix Y*

design matrix X contains an identity submatrix of size m, then the gene expression profiles could be extracted. Flimma now detects this case and rejects such an input, but what about identity submatrices of size less than m?

*Such submatrices reveal individual-level values in XTX when all the other values in the corresponding columns of X are zeroes. This is equivalent to case 3.*

If the design matrix contains two columns that differ only by a single position for example, then one can in fact extract the complete gene expression profile of an individual by subtracting the corresponding rows of the X'Y terms

*A column of all but one zeroes in the design matrix X reveals the expression profile of the sample with a non-zero component. Covariate columns in the design matrix that contain just a single non-zero value are not informative because of the extremely small size of the group defined by this column.*

*A linear combination of any columns in	is equal to the columns of all but one zeroes. This means the presence of almost linearly dependent columns which results in high coefficient variance which is undesirable for linear models. Moreover, to perform this attack, the malicious party should know which linear combination to take.*

What about submatrices that are not identity matrices but are invertible, which can also leak data?

*We do not think that invertible submatrices in X or Y threaten privacy since global X and Y are newer disclosed to any party.*

*We would like to emphasize once again that the proposed list of attack scenarios does NOT pretend to be exhaustive, and we are NOT trying to foresee all possible attacks. We discuss these five attack scenarios in this letter of response only to reject the suspicions that reconstruction attack is easy in the real-world differential expression analysis settings.*

There is also the exceptional case where, with only two parties collaborating, revealing the global update is akin to revealing the local update since each party can guess the other party’s update based on the knowledge of their own.

Existing federated learning solutions typically adopt differential privacy for addressing these leakage concerns, which is not considered.

*As we wrote in the introduction, “differential privacy perturbs the data or results by adding noise to them”. In theory, a hybrid FL-DP tool can guarantee a user-defined privacy level with the cost of accuracy. In practice, finding a meaningful balance between privacy and accuracy is not an easy task. We have tried to combine Flimma with two DP approaches with Laplacian and Gaussian noise models. Unfortunately, we found that both methods greatly affected the results. The plots below compare moderated t-statistics and log-negative p-values obtained by centralized limma-voom and Flimma with DP (ɛ=2).*

*It is important to note that we did not evaluate all the existing DP approaches. We agree with the reviewer, that adding DP is a promising direction for future Flimma development, but further work is needed to identify the most suitable DP approach. Moreover, since DP implies the loss of accuracy compared to the centralized analysis, it should be compared with meta-analyses.*

Lastly, the authors describe in the introduction that “only highly noisy model parameters are shared with the server”. This only describes what each site shares with the aggregator; each site also shares the used noise with the compensator. This is akin to uploading encrypted data to one party and uploading the decryption key to another party. These remaining considerations need to be clarified in the manuscript.

*We added Figure 5 to the Methods section to clarify how HyFed-based Flimma is working and updated the equations in the Workflow section accordingly. As mentioned in the first paragraph in the Discussion, we assume no collusion between the parties and they never exchange the individual noisy parameters or noise values from the clients with each other, and there are at least two clients participating in the study.*

From a practical perspective, HyFed increases the complexity of the system by requiring two independent third parties to play the roles of compensator and aggregator. If these two servers are operated by the same entity, the assumption that the data won’t be combined between these two servers to leak local updates likely won’t be reassuring enough for the users. More detailed explanation of who can play these roles in practice would be helpful.

*We expanded the explanation with:*

*“The aggregator and compensator server components should run on separate machines at distant physical locations. Ideally, to minimize the risk of reconstruction attacks, they should be controlled by trusted third-party organizations not connected to any of the study participants. Currently, the publicly available Flimma webtool is using the aggregator running at the Chair of Experimental Bioinformatics, Technical University of Munich (Germany) while the compensator is hosted at the Department of Mathematics and Computer Science at the University of Southern Denmark (Denmark).”*

**Fourth round of review**

**Reviewer 2**

In response to the comment that the security of HyFed needs to be better justified, the authors claimed that “HyFed implements a well-known additive secret sharing whose privacy proof has already been provided by the corresponding studies such as Shamir 1979”. This is misleading for two reasons. First, the cited Shamir secret sharing scheme based on polynomial interpolation is fundamentally different from what HyFed implements. Second, the manuscript’s description of “noise” added in HyFed in Methods is presented in an informal language that does not clarify its connection to standard secret sharing schemes, which is why this concern was initially raised. Proof of perfect privacy for additive secret sharing requires that the numbers be represented as elements in a ring or a finite field. In this work’s setting, the data values are real numbers, and the noise is sampled as 32-bit integers. It is not clear at all how the authors are interpreting these as secret shares, which makes it difficult to assess whether the scheme is designed appropriately.

The paper does not draw a fair distinction between Flimma’s FL approach vs. HE or SMPC approaches. In the added text in Introduction, all three are described as “privacy-aware” not “privacy-preserving” but the latter two clearly provide stronger guarantees than FL. The description overlooks the differences between leakage in the final output (risk shared by all three -- could be considered unavoidable by some) vs. leakage in the intermediate results.

In Discussion the authors draw a misleading equivalence between the remaining privacy risks of Flimma and meta-analysis approaches. This similarly overlooks the fact that Flimma shares intermediate results that are typically higher dimensional and more prone to potential leakage than meta-analysis.

**Reviewer** **3**

The proposed approach, though not perfect in its privacy guarantees, is an improvement relative to current approaches (e.g. meta-analysis). That said, there is a bigger concern (also articulated by the reviewer): the lack of details about the proposed HyFed scheme. This detail is also missing from the preprint on HyFed. While the theoretical properties of the secret sharing scheme are indeed well-known, there are non-trivial issues in mapping GWAS and DGE analyses to the federated setting while being privacy aware. As one simple but important example, the secret sharing schemes such as the one that the authors refer to and rely on assume the data is finite e.g. integer-valued while the GWAS/DGE data are real-valued. Handling this gap is an important technical challenge that can potentially impact the privacy and/or the accuracy of the method. I could not find any clear description/discussion of this issue. In my mind, this is a serious gap in the submission.

**Authors Response**

**Point-by-point responses to the reviewers’ comments:**

Reviewer 3:

The proposed approach, though not perfect in its privacy guarantees, is an improvement relative to current approaches (e.g. meta-analysis). That said, there is a bigger concern (also articulated by the reviewer): the lack of details about the proposed HyFed scheme. This detail is also missing from the preprint on HyFed. While the theoretical properties of the secret sharing scheme are indeed well-known, there are non-trivial issues in mapping GWAS and DGE analyses to the federated setting while being privacy aware. As one simple but important example, the secret sharing schemes such as the one that the authors refer to and rely on assume the data is finite e.g. integer-valued while the GWAS/DGE data are real-valued. Handling this gap is an important technical challenge that can potentially impact the privacy and/or the accuracy of the method. I could not find any clear description/discussion of this issue. In my mind, this is a serious gap in the submission.

*The current version of HyFed treats non-negative integer-valued and real-valued parameters differently. For non-negative integers, it employs the standard additive secret sharing scheme based on finite field arithmetic (Cramer et al., 2015). For matrices with floating-point numbers and negative integers, HyFed uses a real number secret sharing approach based on Gaussian distribution (Dibert & Csirmaz, 2014; Tjell & Wisniewski, 2021). We added an additional subsection explaining the masking scheme, the choice of parameters, and their effect on privacy and accuracy.*

*Besides the changes recommended by the reviewers and the Editor, we*

*-	added structure to Discussion, now it is split into two subsections;*

*-	moved the figure illustrating batch effects in the data from supplementary to the main text;*

*- slightly rewritten Abstract and Conclusion, without the addition of any new statements;*

*- modified Fig.1 labels (“federated analysis” changed to “hybrid federated analysis” and captions for privacy-awareness and robustness to imbalance are added) and Fig. 6 labels and captions to match with the new section on masking scheme.*

*All changed parts of the paper are highlighted with the red text color.*

Reviewer 2:

In response to the comment that the security of HyFed needs to be better justified, the authors claimed that “HyFed implements a well-known additive secret sharing whose privacy proof has already been provided by the corresponding studies such as Shamir 1979”. This is misleading for two reasons. First, the cited Shamir secret sharing scheme based on polynomial interpolation is fundamentally different from what HyFed implements. Second, the manuscript’s description of “noise” added in HyFed in Methods is presented in an informal language that does not clarify its connection to standard secret sharing schemes,

which is why this concern was initially raised. Proof of perfect privacy for additive secret sharing requires that the numbers be represented as elements in a ring or a finite field. In this work’s setting, the data values are real numbers, and the noise is sampled as 32-bit integers. It is not clear at all how the authors are interpreting these as secret shares, which makes it difficult to assess whether the scheme is designed appropriately.

*The updated version of HyFed implements two different ways of random number generation depending on the input matrix data type. The new masking scheme is explained in an additional subsection.*

The paper does not draw a fair distinction between Flimma’s FL approach vs. HE or SMPC approaches. In the added text in Introduction, all three are described as “privacy-aware” not “privacy-preserving” but the latter two clearly provide stronger guarantees than FL.

*We updated the definition of privacy-preserving approach:*

*“We call such a privacy-aware approach privacy-preserving (e.g. DP), if it provides a formally proven privacy guarantee that captures the risks associated with each sample of the dataset.”*

*We also clarified the advantage of SMPC and HE in Introduction:*

*“However, the approaches based on pure HE and SMPC provide stronger privacy compared*

*to FL-based approaches because they reveal less information to the third parties.”*

The description overlooks the differences between leakage in the final output (risk shared by all three -- could be considered unavoidable by some) vs. leakage in the intermediate results.

*We did not discuss the risk of attacks at the final output because none of the methods and studies that make the final results public can avoid this risk. Therefore we only discuss the risks of attacks at intermediate local and global parameters. Since we assume honest-but-curious parties and local parameters are protected by noise, we discuss reconstruction attacks on global intermediate parameters. We also added the assumption that the number of clients is larger than two to exclude the possibility to recover local parameters belonging to the one client by the second client.*

In Discussion the authors draw a misleading equivalence between the remaining privacy risks of Flimma and meta-analysis approaches. This similarly overlooks the fact that Flimma shares intermediate results that are typically higher dimensional and more prone to potential leakage than meta-analysis.

*It was not our intention to claim the equivalence of risks. We highlight that meta-analysis, the only existing practical (not theoretical, like a hypothetical DP or HE-based tool) competitor of Flimma, also does not guarantee perfect protection from reconstruction attacks. We reformulated this part and highlighted differences w.r.t. potential reconstruction attacks:*

*Note that the risk of reconstruction attack is not excluded for meta-analysis methods. Although local p-values and effect sizes appear to be less prone to reconstruction attack than the aforementioned intermediate global parameters computed by Flimma, no formal proof of this intuition is provided. Despite that, meta-analyses remain popular approaches that are not in conflict with privacy legislation. In addition to a reasonable protection of the raw data, Flimma offers better accuracy than meta-analysis methods.*

**Fifth round of review**

**Reviewer 3**

I am satisfied with the author reponses now. The claims of privacy are toned down and accurate now.
